# Biobased Interpenetrating Polymer Network Membranes for Sustainable Molecular Sieving

**DOI:** 10.1021/acsnano.3c10827

**Published:** 2024-02-20

**Authors:** Joyce Cavalcante, Diana G. Oldal, Maxim V. Peskov, Aron K. Beke, Rifan Hardian, Udo Schwingenschlögl, Gyorgy Szekely

**Affiliations:** † Advanced Membranes and Porous Materials Center, Physical Science and Engineering Division (PSE), King Abdullah University of Science and Technology (KAUST), Thuwal 23955-6900, Saudi Arabia; ‡ Materials Science and Engineering Program, Physical Science and Engineering Division (PSE), King Abdullah University of Science and Technology (KAUST), Thuwal, 23955-6900, Saudi Arabia; § Chemical Engineering Program, Physical Science and Engineering Division (PSE), King Abdullah University of Science and Technology (KAUST), Thuwal, 23955-6900, Saudi Arabia

**Keywords:** biopolymer, membrane, nanofiltration, solvent, latex, agarose

## Abstract

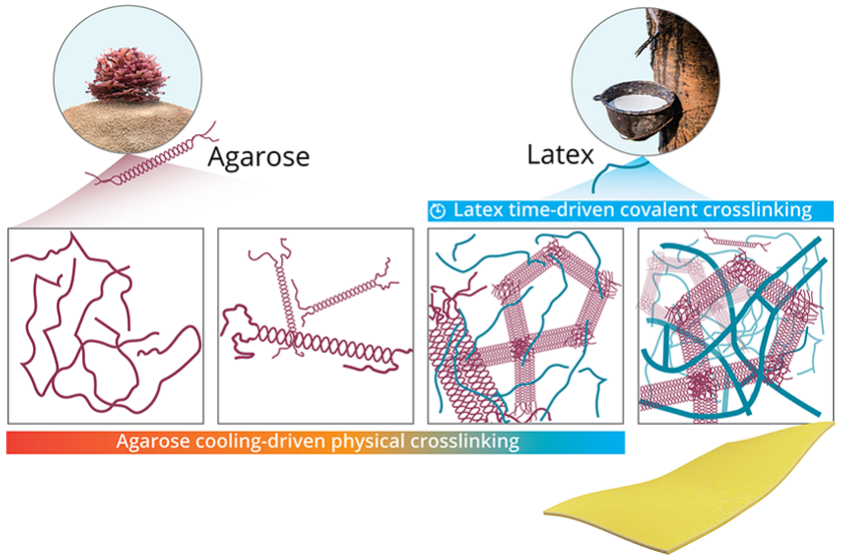

There is an urgent need for sustainable alternatives to fossil-based polymer materials. Through nanodomain engineering, we developed, without using toxic cross-linking agents, interpenetrating biopolymer network membranes from natural compounds that have opposing polarity in water. Agarose and natural rubber latex were consecutively self-assembled and self-cross-linked to form patchlike nanodomains. Both nano-Fourier transform infrared (nano-FTIR) spectroscopy and computational methods revealed the biopolymers’ molecular-level entanglement. The membranes exhibited excellent solvent resistance and offered tunable molecular sieving. We demonstrated control over separation performance in the range of 227–623 g mol^–1^ via two methodologies: adjusting the molecular composition of the membranes and activating them in water. A carcinogenic impurity at a concentration of 5 ppm, which corresponds to the threshold of toxicological concern, was successfully purged at a negligible 0.56% pharmaceutical loss. The biodegradable nature of the membranes enables an environmentally friendly end-of-life phase; therefore, the membranes have a sustainable lifecycle from cradle to grave.

## Introduction

Organic solvent nanofiltration (OSN) is a membrane process that is used for molecular separation in harsh organic media, which benefits multiple industries (e.g., petrochemical, biorefining, paint, pharmaceutical, and food).^1^ This pressure-driven separation technology is conducted by combining solute-size-level sieving and the solution/diffusion mechanisms. OSN uses membranes that are stable in organic solvents to separate solutes in the molecular range of 100–2000 g mol^–1^.^2^ Nevertheless, the fabrication of an OSN membrane remains considerably challenging in terms of selecting sustainable solvents and materials. Currently, conventional OSN membrane fabrication relies on petrochemical-derived polymers and monomers, as well as toxic solvents,^3^ which undermines the achievement of the United Nations Sustainable Development Goals, particularly Goal 11 (Sustainable Cities and Communities), Goal 12 (Responsible Consumption and Production), and Goal 13 (Climate Action).

The emerging need for sustainable solutions, such as the application of green solvents and biobased material selection, is mobilizing the scientific community to develop holistic cutting-edge strategies for membrane manufacture.^4^ Derivatives of natural polymers have been used since the introduction of membrane-based separations.^5^ However, the processing of such materials typically requires the application of harsh solvents and toxic chemicals.^6^ More sustainable solvents, such as ionic liquids, have been used to process biobased membranes; however, these solvents remain expensive.^1,7^ Hence, the need for fully sustainable OSN systems, designed considering a closed-loop lifecycle and cost-effectiveness, still presents key challenges.

**Figure 1:**
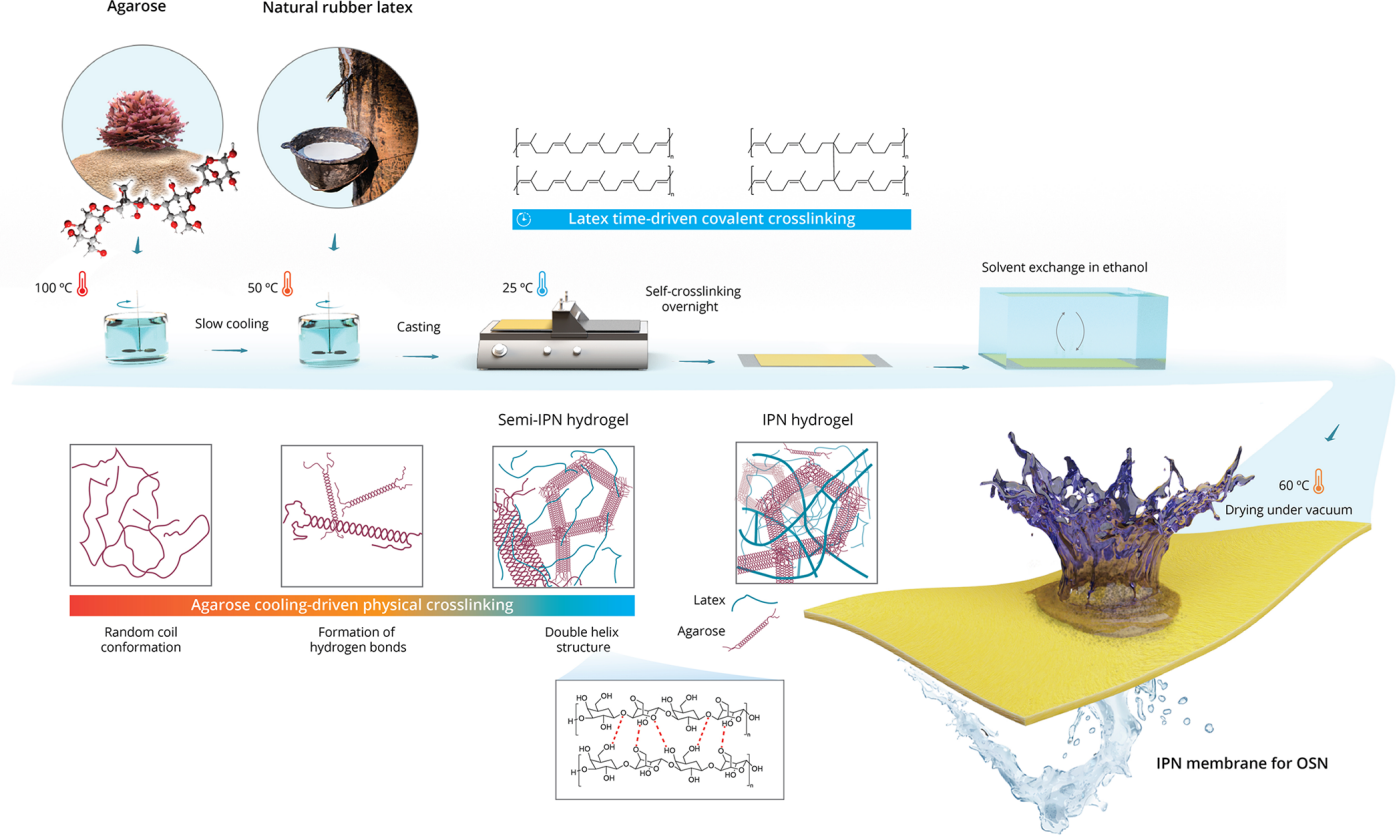
Schematic of the explored IPN fabrication route. Dissolution of agarose in water was followed by the addition of latex above the gelation point of agarose for adequate mixing at the molecular level. The solution was cast at room temperature, and agarose was physically cross-linked by the formation of hydrogen bonds, where the random-coiled agarose chains were transformed into a solid network of double helix structures. The semi-IPN hydrogel became an IPN system after the overnight cross-linking of latex. Solvent exchange in ethanol was conducted, and the IPN gel films were dried in IPN membranes for organic solvent nanofiltration.

Polymer cross-linking is often required to increase the thermochemical stability of nanofiltration membranes in harsh organic media. However, the majority of cross-linking agents used in polymeric membranes for OSN exhibit alarming toxicity. Because cross-linking agents are reactive species, they usually exhibit cytotoxicity, genotoxicity, mutagenicity, and carcinogenicity, which pose a threat to both humans and the environment.^8^ Commonly used cross-linkers for fabricating OSN membranes that pose toxicity risks are aldehydes (e.g., acetaldehyde and glutaraldehyde),^2^ dimethacrylates (e.g., ethylene glycol dimethacrylate and diethylene glycol dimethacrylate),^9^ diamines (e.g., *N*,*N*,*N*′,*N*′-tetramethylethylenediamine and *N*,*N*,*N*′,*N*′-tetramethyl-1,6-hexanediamine),^10^ and chlorinated compounds (e.g., epichlorohydrin).^11^ Thus, an alternative solvent-resistant membrane that is made from natural materials without the addition of reactive species is highly desired (Figure 1).

Interpenetrating polymer networks (IPN) are a class of polymer materials comprising two or more polymeric systems that are self-cross-linked and interlaced at the molecular level. IPN systems, particularly those derived from natural materials (BioIPN), are a promising alternative to conventional film materials for use in coatings, packaging, and the use of OSN membranes. Biodegradable IPN hydrogels made from prevulcanized natural rubber and cassava starch have been reported as coating membranes for the slow release of fertilizers.^12^ Moreover, IPN membranes based on cellulose/agarose hydrogel systems have been developed as a support for aqueous electrolytes.^13^ Agarose is a renewable, nontoxic, biodegradable, and low-cost polymer. However, to the best of our knowledge, no studies have been conducted in which a naturally derived agarose-based IPN has been used for the OSN. The main drawback of using IPN for the OSN is related to the highly dense structures that form in the IPN upon cross-linking. Therefore, a semi-IPN membrane that offers the advantages of an IPN while still exhibiting porosity is a suitable alternative for OSN applications.^14,15^ IPNs provide better stability than semi-IPNs due to the simultaneous formation of multiple interconnected polymer networks. Their improved dimensional stability reduces the likelihood of deformation or shrinkage under varying conditions. Moreover, the simultaneous presence of two distinct polymers in IPNs can lead to enhanced chemical resistance and thermal stability. In this study, we investigated the formation of IPN by combining agarose and natural latex (Table 1). An interlaced IPN was fabricated by combining the temperature-driven physical cross-linking of agarose and the time-driven covalent cross-linking of latex.

**Table 1:** Designations of Membranes According to the Conditions of Fabrication and Composition^b^

membrane	*C*_agarose_ (wt %)	*C*_latex_ (wt %)	activation (s)
agarose^a^	100	0	0
latex^a^	0	100	0
BioIPN^0^	90	10	0
BioIPN^10^	90	10	10
BioIPN^15^	90	10	15
BioIPN^20^	90	10	20

a Benchmark membranes.

b Ethanol was used as the nonsolvent in a coagulation bath for solvent exchange, and a vacuum of 30 mbar was used to dry the membranes.

Agarose is a nonionic linear polysaccharide extracted from red seaweeds from the Rhodophyceae class and is one of the two main components of agar, in addition to agaropectin. It consists of repeating units of d-galactose and 3,6-anhydro-l-galactopyranose alternating units that are linked via α-(1,3) and β-(1,4) glycosidic bonds and possess polar OH groups.^16^ Agarose has been widely used in agribusiness, the pharmaceutical industry, and as an electrophoresis medium, because of its ability to form a hydrogel network via thermoreversible polysaccharide gelation.^17^ Natural rubber latex, also known as poly(*cis*-1,4-isoprene), is a bioelastomer that is extracted from the Hevea Brasiliensis tree,^18^ which grows in several tropical countries such as Brazil, Thailand, and Malaysia. The flexibility and biocompatibility of latex make it suitable for use in the production of gloves, dental dams, pacifiers, seals, automobile parts, and drug delivery membranes.^19,20^ Furthermore, the versatility of latex stems from its physical properties, nonpolar hydrocarbon structure, renewability, sustainability, processability, and low cost.^21^ The mechanical properties of latex are attributed to the formation of a state of low cross-linking density upon film formation.^22^ The opposing polarity of latex and agarose biopolymers presents a chemical incompatibility challenge that is resolved by the findings of this study through an investigation of molecular-level interactions.

Herein, we investigated the physicochemical properties of BioIPN membranes fabricated from agarose and natural rubber latex. Furthermore, membrane activation with water was conducted. State-of-the-art characterization techniques including cryoelectron microscopy and nano-Fourier transform infrared (nano-FTIR) spectroscopy were employed to study the morphology and chemical information on the membranes at the nanoscale. Moreover, the effect of the OSN via crossflow was evaluated for membranes fabricated under multiple processing conditions to understand their effect on the OSN performance. The applications of the fabricated IPN membranes were tested for active pharmaceutical ingredient (API) purification to remove carcinogenic impurities. Furthermore, the biodegradability of the fabricated IPN membranes was assessed to ensure the end-of-life sustainability of the membranes.

## Results and Discussion

### Physicochemical Characteristics of the Membrane

**Figure 2:**
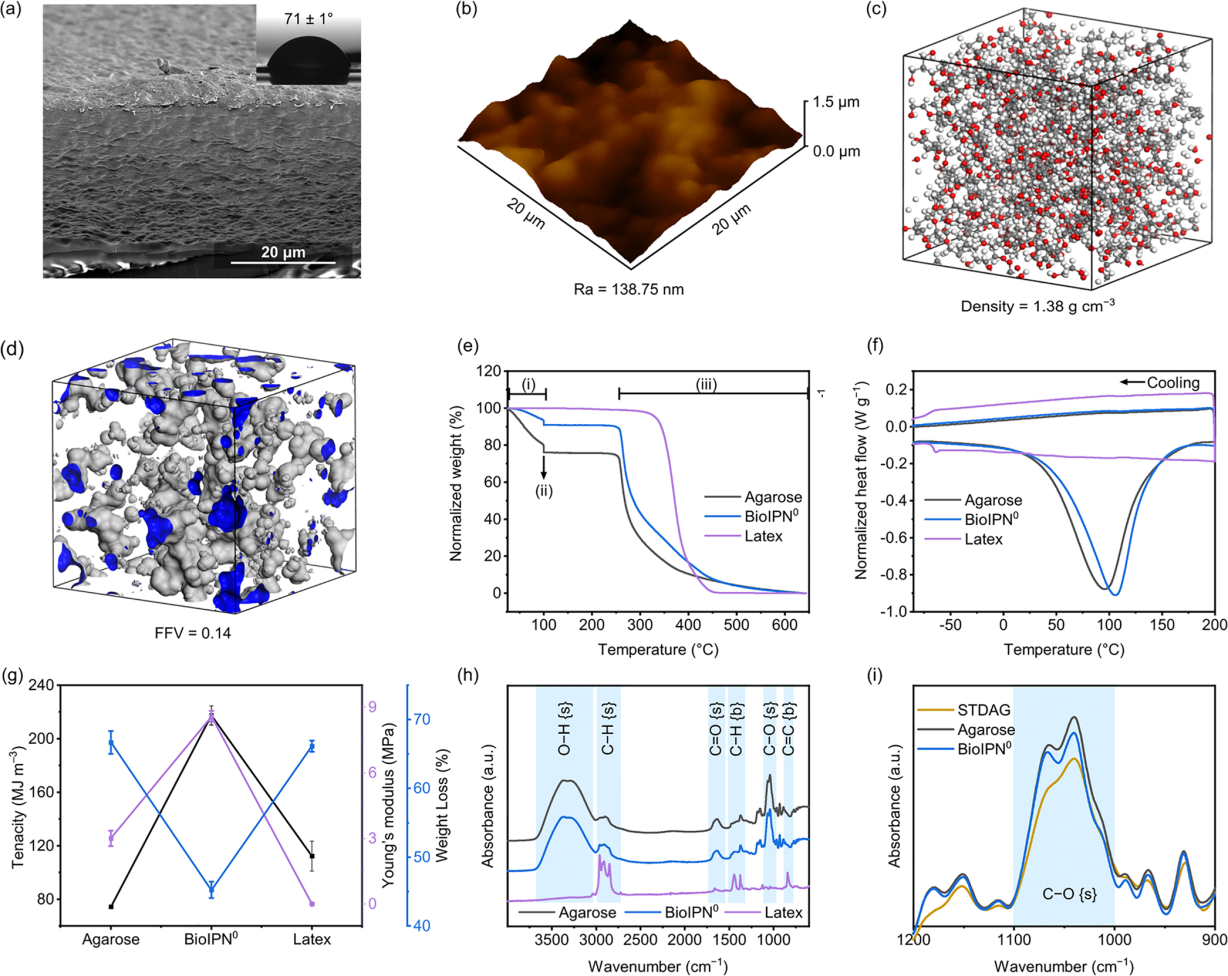
Physicochemical properties of the membranes. (a) SEM cross-section and top surface of BioIPN^0^, with WCA presented as an inset. (b) AFM three-dimensional projection. (c) Molecular simulation of polymer packing and (d) FFV for BioIPN^0^ [legend: gray spheres represent C atoms, red spheres represent O atoms, and white spheres represent H atoms]. (e) TGA and (f) DSC spectra of the membranes fabricated in this study. (g) Tensile properties obtained via mechanical characterization of the investigated membranes and biodegradation analysis of membrane systems considering weight loss due to enzymatic treatment. Chemical characterization via (h) FTIR spectroscopy of the fabricated membranes. The {s} and {b} designations correspond to the peaks caused by the stretching and bending modes, respectively. (i) Expanded view of the FTIR spectrum in the alcoholic C–O {s} region.

The morphology of the fabricated BioIPN^0^ was investigated via scanning electron microscopy (SEM) (Figure 2a). Both cross-sectional and top surface images revealed a texturized dense microstructure with a water contact angle (WCA) of 71° ± 1°. Atomic force microscopy (AFM) measurements (Figure 2b) revealed a roughness value (*R*_a_) of 138.75 nm, and the molecular simulation of the polymer packing in the BioIPN^0^ correlated a density of 1.38 g cm^–3^ (Figure 2c) to a fractional free volume (FFV) of 0.14 (Figure 2d). The pure agarose membrane was found to be considerably dense (Figure S8a). The addition of latex imparted greater flexibility to the system, which facilitated rapid solvent removal upon vacuum drying and resulted in texturized membranes. As anticipated, no relevant change was observed in the quantitative analysis of the membrane thickness (Figure S9).

The thermogravimetric analysis (TGA) plot shown in Figure 2e confirms that BioIPN^0^ exhibited better thermal behavior than agarose as the weight loss of agarose was observed to be greater than that of BioIPN^0^ for temperatures of <450 °C. In accordance with the TGA results, the differential scanning calorimetry (DSC) peak (Figure 2f) that corresponded to the glass-transition temperature (*T*_g_) of the BioIPN^0^ was ∼10 °C higher than that of pristine agarose (Table S4). An increase in the *T*_g_ value corroborates the TGA findings that the IPN exhibited better thermal stability than the pristine systems, since higher thermal energy was required by the polymer chains to achieve mobility during heating. Furthermore, BioIPN^0^ presented superior values for both tenacity and Young’s modulus (Figure 2g), proving that the optimum mechanical features were achieved using a formulation of 90 wt % agarose with 10 wt % latex. The time-driven covalent cross-linking within BioIPN^0^ was confirmed via mechanical analysis (Table S5). The pure latex system, which was processed under the same fabrication conditions as BioIPN^0^, presented a substantial maximum elongation value (>200%), which is indicative of an elastomeric behavior with low cross-linking density. By comparison, the BioIPN systems demonstrated elongation values between 4% and 10%, suggesting a high cross-linking density.

Because both agarose and latex are biodegradable, our BioIPN also offered this advantage, as confirmed by the subsequent agarase–laccase enzymatic treatment of BioIPN^0^. The lower weight loss of BioIPN^0^, in comparison with its pristine counterparts (Figure 2g), may be attributed to its high mechanical stability, which results from the formation of anchoring points in the IPN. The biodegradability of various BioIPN systems against a negative polypropylene control was tested and confirmed over a period of 14 days, demonstrating their ability to break down naturally (Figure S6d). Our results indicate that a combination of biodegradable polymers in an IPN system can preserve the biodegradability of the polymer system as a whole. These findings facilitate exploring biodegradable IPN systems for various applications, including but not limited to membranes.

Attenuated total reflection Fourier transform infrared spectroscopy (ATR-FTIR) suggests that the C–H bending peaks observed at ∼1380 cm^–1^ for BioIPN^0^ (Figure 2h) originated from the C–H bonds in both latex and agarose. Similarly, the C–H stretching peaks in the range of 3000–2840 cm^–1^ were attributed to the alkane groups in both the latex and agarose chemical structures, which were identified in all cases. The C=C bending peaks observed at ∼810 cm^–1^ highlight the presence of unsaturated double bonds on the core of the *cis*-1,4-polyisoprene repeating unit of the latex. This peak was not present in any of the other investigated systems. It was exclusively detectable in the pristine latex material. The alcoholic C–O stretching peaks (at ∼1040 cm^–1^) for BioIPN^0^ (Figure 2i) were more intense than for the non-cross-linked commercial agarose powder (STDAG), suggesting that cross-linking was achieved via the formation of a new bond (C–O–C) under the selected processing conditions. Both FTIR spectroscopy and mechanical analysis were fundamental to concluding that both components of the presented IPN systems were indeed cross-linked. Moreover, the physical cross-linking of agarose was confirmed via the practical observation of hydrogel formation upon the cooling of all the IPN systems (Figure S1).

**Figure 3:**
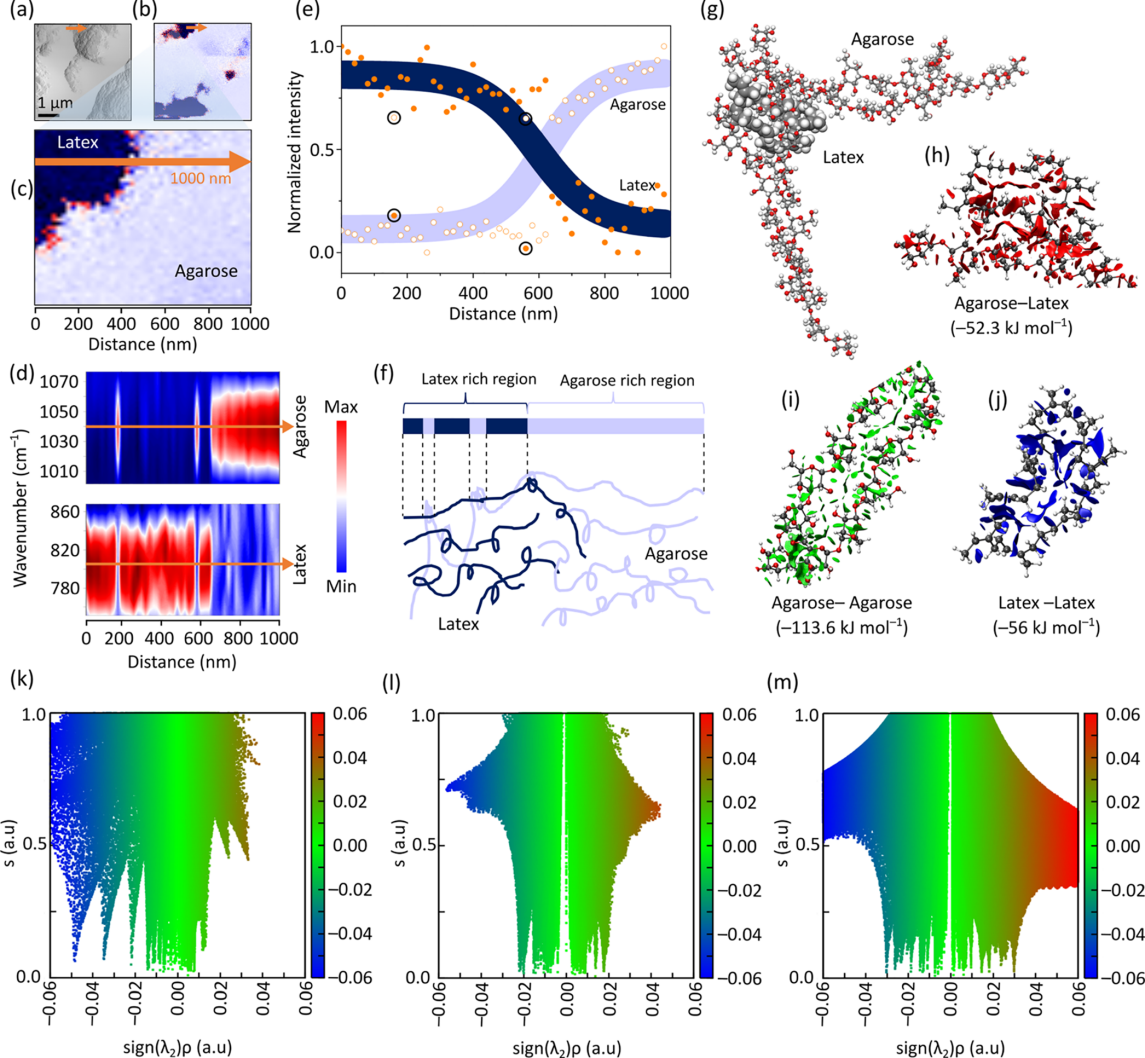
Nanodomain analysis and computational simulations of the BioIPN. (a) AFM topography of the BioIPN^0^ membrane. (b) Phase image of the corresponding AFM topography. (c) Magnification of the phase image in Figure 1b. Color contrast (dark blue and light blue) indicates different chemistry; orange arrow indicates the nano-FTIR scanning line. (d) Contour plot image of the nano-FTIR intensity profile with the characteristic vibration of C=C from latex (810 cm^–1^) corresponding to the dark blue area in Figure 1c, and with the characteristic vibration of C–O from agarose (1040 cm^–1^) corresponding to the light-blue area in Figure 1c. (e) Intensity profile of latex and agarose along the scanning line. Closed orange circles indicate the intensities for latex (peak position at 810 cm^–1^) and open circles indicate the intensities for agarose (peak position at 1040 cm^–1^). Black circles indicate the presence of agarose in the latex-rich region. Nano-FTIR spectroscopy was performed by using a spectral resolution of 33 cm^–1^. (f) Illustration of the entanglement between latex and agarose derived from Figures 1d and 1e: on the membrane surface, some agarose polymer was observed in the latex-rich region. (g) Geometries, interaction energies, and noncovalent interaction analysis of (h) agarose–latex, (i) agarose–agarose, and (j) latex–latex in the BioIPN^0^ membrane. (k–m) Two-dimensional diagrams of the reduced density versus the product of the charge density and the sign of the second Hessian eigenvalue λ_2_ for the agarose–agarose interactions (panel (k)), agarose–latex interactions (panel (l)), and latex–latex interactions (panel (m)). The colors show the relative strength of the noncovalent interaction over a range from attractive (blue) to repulsive (red).

A characterization technique that can distinguish between the chemistries of agarose and latex at the nanoscale is required to conduct structural analysis of the BioIPN^0^ membrane. Standard ATR-FTIR is constrained by low spatial resolution (micrometer size) that limits access to the chemical information at the nanoscale. To overcome this limitation, we performed nano-FTIR spectroscopy, which combines AFM and FTIR spectroscopy to reveal the chemical information at nanometer spatial resolution (Figure 3).

The AFM topography of BioIPN^0^ indicated that it had a textured surface (Figure 3a). Interestingly, although the IPN membrane seemed to be homogeneous macroscopically, it showed a polymer domain at nanoscale. Thanks to the nano-FTIR spectroscopy technique, this variability in the nanoscale can be visualized. The corresponding phase image of the AFM topography showed a contrast that indicated the presence of domains having different chemistries (Figure 3b). To uncover the chemistry of the nanodomains, a line scan was performed along the orange arrow shown in Figure 3c. The peak intensities along the scanning line are presented as contour plots (Figure 3d), where the red and blue colors indicate the maximum and minimum intensities, respectively. In Figure 3d, the maximum intensities (at ∼810 cm^–1^, which is characteristic for latex) were observed up to ∼600 nm within the scanning line, indicating that the dark blue regions in Figure 3b and Figure 3c are attributed to latex. In Figure 3d, the maximum intensities (at ∼1040 cm^–1^, which is characteristic for agarose) were observed from 600 to 1000 nm within the scanning line, suggesting that the light-blue regions in Figure 3b and Figure 3c are ascribed to agarose. Interestingly, strong blue lines were observed in the red region of Figure 3d (agarose), and strong red lines were also observed in the blue region of Figure 3d (latex). These observations suggest that a small portion of the agarose spectra was identified in the latex-rich area, indicating entanglement between agarose and latex (Figure 3f). The intensity profile along the scanning line (derived via normalization of the nano-FTIR peak intensity) is also represented in Figure 3e. Initially, only the maximum peak intensities of latex (dark blue) were observed; these intensities started to decline at ∼600 nm within the region indicated by the scanning line. Similarly, the peak intensities of agarose (light blue) were initially not apparent and then started to appear with diminishment of the latex regions.

To support the observation by nano-FTIR spectroscopy, density functional theory (DFT) and molecular dynamics (MD) simulations were performed to reveal the molecular-level interactions among the polymer constituents of BioIPN^0^ in water, which was used as the membrane preparation medium (Figure 3g). Noncovalent interaction analyses were executed for agarose–latex (Figure 3h), agarose–agarose (Figure 3i), and latex–latex (Figure 3j) polymer chains. The DFT binding energies of the polymer pairs were −52.3, −113.6, and −56 kJ mol^–1^, respectively. These strong interactions within the polymer pairs explain the existence of the three regions observed in Figures 3d and 3e. Interestingly, agarose interacts with latex through its hydrophobic components (CH_2_ and CH) by weak van der Waals forces. The hydrophobic latex does not establish hydrogen bonds with water and minimizes its energy by intramolecular latex–latex interactions. Energy-favorable increase in the number of such interactions lead to folding of the latex chain into a compact conformation with ellipsoidal shape (see Figure 3g, as well as Figures S21 and S22). For good shielding of the folded latex chain from water molecules in the environment—which minimizes the energy of the system—several agarose chains are needed to cover a wide area around the folded latex chain. Our simulations show that agarose acts similarly to a surfactant by isolating latex from water.

An analysis of the type and strength of the noncovalent interactions between the polymers was performed using diagrams of the reduced density (*s*) versus sign(λ_2_)ρ (Figures 3k–m). All three plots feature a cluster of troughs at low density (green color), where the reduced charge density is close to zero, indicating the presence of van der Waals interactions between the polymer chains. A wider distribution indicates a larger number of van der Waals interactions (such as H**···**H and H**···**C) within the latex chain as well as between the latex and agarose chains than between the agarose chains. The agarose chains are bound by strong hydrogen bonds (blue color in Figure 3k, where the reduced charge density is close to zero) provided by the hydroxyl groups, as shown by the three troughs of *s* in the region [−0.05; −0.02] of sign(λ_2_)ρ. The trough positions indicate that the hydrogen bonds have different strengths.

### Membrane Separation Performance

**Figure 4:**
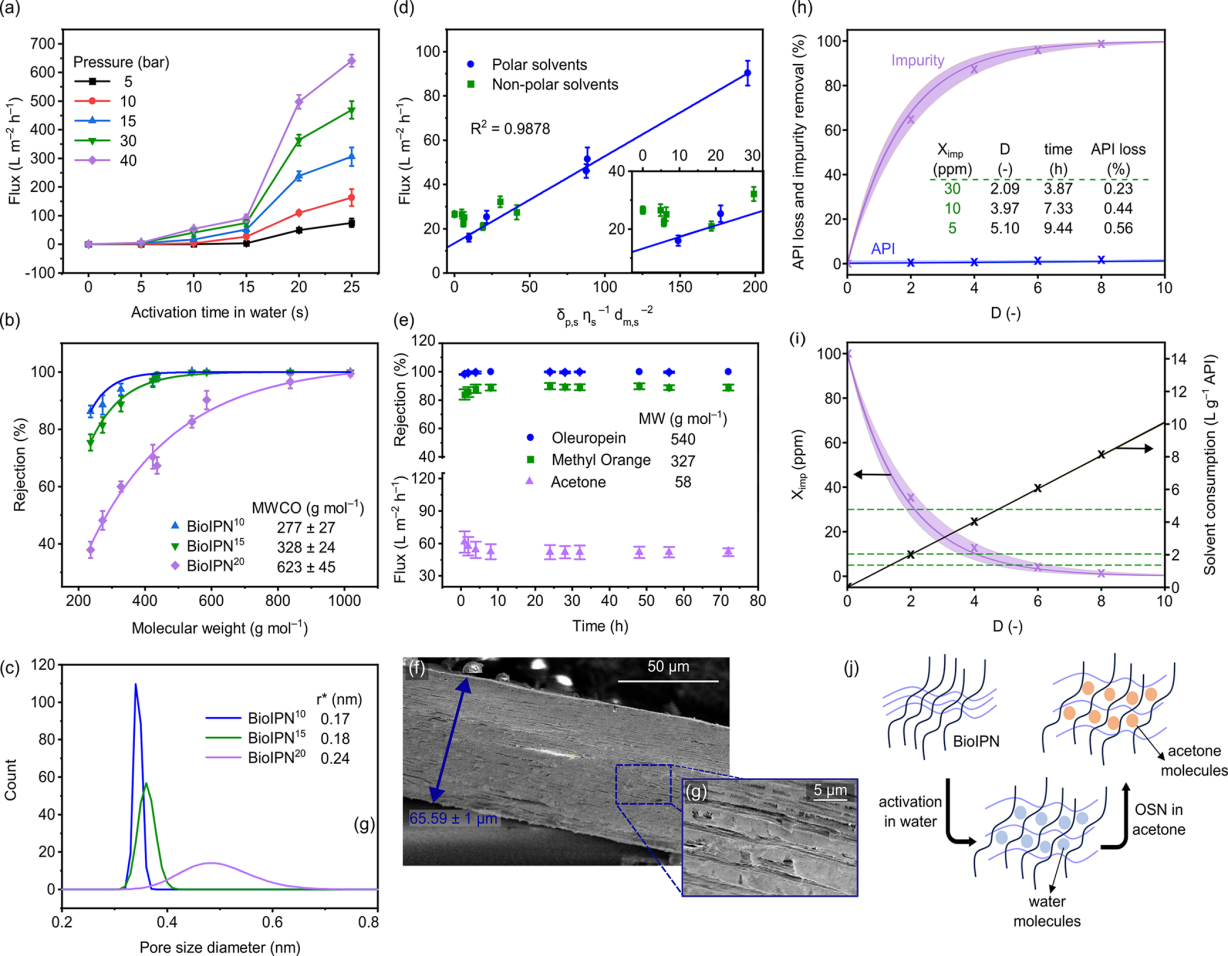
Nanofiltration performance of IPN membranes. (a) Solvent flux through BioIPN^0^ after being activated in water for various lengths of time, investigated under multiple pressures. (b) Rejection profiles and MWCO values for BioIPN after being activated in water for various lengths of time, investigated at 20 bar. (c) Pore size distribution of BioIPN after being activated in water for various lengths of time. (d) Pure solvent flux profile as a function of the solubility parameter for BioIPN^15^ at 20 bar. (e) Flux and rejection profiles presenting long-term stability of the performance of BioIPN^15^ at 20 bar. Acetone was used as the solvent for nanofiltration, unless otherwise stated. (f, g) Cross-section cryo-SEM image. (h, i) API loss, impurity removal, impurity ratio, and solvent consumption for BioIPN^15^, as a function of the number of diavolumes. Measured data points are included for the BioIPN^15^ membrane. The borders of the shaded regions correspond to the 25^th^ and 75^th^ percentiles of the Monte Carlo simulations. (j) Proposed scheme of the structural evolution of IPN during activation in water and the performance of the OSN performance.

BioIPN^0^ was used to study the correlation between solvent flux and activation time in water (Figure 4a) because this membrane displayed the most uniform morphology among other membranes fabricated with different compositions (see Figures S8–S10). For all of the cases, no acetone flux was observed without activating the membranes in water (0 s). At 5 bar, the acetone flux was only observed after 15 s of activation. Overall, moderate acetone flux was observed for 10, 15, 20, and 25 s of activation. These observations indicate that a combination of pressure and activation time is necessary for a gradual increase in the solvent flux. We hypothesize that the polar characteristic of water molecules may penetrate the hydrophilic BioIPN and slightly rearrange the polymeric network, creating the pores in the BioIPN. Activation in water for longer than 25 s resulted in membranes with a virtually infinite flux; therefore, these membranes were excluded from further filtrations.

The molecular weight cutoff (MWCO) values of the IPN systems gradually increased from 227 ± 27 (in BioIPN^10^) to 623 ± 45 g mol^–1^ (in BioIPN^20^) as the activation time in water increased from 10 to 20 s (Figure 4b), indicating that activation time in water can be used to control nanofiltration performance. The increase in MWCO values was consistent with the increase in the pore diameter of the membrane calculated for all scenarios; furthermore, 10, 15, and 20 s of activation in water led to pore diameters of 0.34, 0.36, and 0.48 nm, respectively (Figure 4c). Prolonging the activation time (for example, 25–60 s) led to rejection curves below 90%, thus neither the MWCO nor the pore size values could be estimated. The pore sizes were estimated from the rejection curves, which were obtained using neutral molecules as solutes such as polystyrenes with various molecular weights. The molecular size of those polystyrenes can be correlated with their diffusivity and molecular size (solute molar volume and Stokes radius) as detailed in section S8 in the Supporting Information.

A linear correlation (*R*^2^ = 0.9878) for polar solvents was observed between the pure solvent flux and the solubility parameter (Figure 4d), which consisted of the solvent solubility (δ_p,s_), viscosity (η_s_), and molar diameter (*d*_m,s_) (see Table S7). The majority of the investigated nonpolar solvents exhibited a relatively high flux, compared with that predicted via linear interpolation (inset of Figure 4d). The variance in the flux averages for the polar solvents was 63 times higher than that for the nonpolar solvents (Table S7). The polar solvents generally showed a higher flux than nonpolar solvents. These findings indicated that since the BioIPN is hydrophilic (due to the hydroxyl group from agarose), the membranes had better wetting degrees with polar solvents, facilitating faster flow and resulting in higher flux. Thus, membrane performance can be used with optimum linear control over polar solvents.

The long-term stability of BioIPN^15^ was evaluated during a continuous crossflow filtration over 72 h (Figure 4e). An API, i.e., oleuropein (540 g mol^–1^), and a dye, i.e., Methyl Orange (327 g mol^–1^), were used as the solutes. The rejection value of Methyl Orange was found to be 88.1% ± 2.5%, which was 11.4% lower than that of oleuropein (99.5% ± 0.6%). The observed difference in the rejection value can be attributed to the lower molecular weight of Methyl Orange. Filtration in acetone resulted in a stable average flux of 53 ± 5.8 L m^–2^ h^–1^ over 72 h. The standard deviation values of the long-term study decreased over time (Figure S14), which was due to the initial compaction and stabilization of the nanofiltration system (commonly observed). The nanofiltration test demonstrated that both the flux and the rejection profiles were stable.

To further investigate the evolution of the BioIPN^15^ microstructure during nanofiltration, cryo-SEM was carried out to demonstrate the changes after the 15 s activation time in water (Figures 4f and 4g). An accurate prediction for the degree of swelling (∼158% ± 1%) was then obtained by comparing the thickness values of the IPN before (BioIPN^0^) and after (BioIPN^15^) the 15 s of activation in water (Figures S19b–S19c). The swelling in water was responsible for opening up the microstructure of BioIPN^0^ by generating microvoids (Figure 4j), which allowed a higher solvent flux through the membrane. Therefore, without the activation step, no flux was observed (Figure 4a).

Assuming isotropic swelling, a molecular simulation was performed to predict the FFV of BioIPN^15^ (Figure S20). The model takes into account the swelling of BioIPN^15^ upon activation and forecasts an increase in the FFV to 0.5, in comparison to the results obtained for dry BioIPN (FFV = 0.14). The simulation was derived from the information on the membrane’s thickness obtained from the presented SEM techniques (performed at both cryogenic and room temperatures). The simulation details can be found in Figures S19 and S20. The dynamics of our nanofiltration results establish a fresh perspective on the application of dense, naturally derived IPN membranes in nanofiltration systems. Biopolymers can be fine-tuned to fit the requirements of the OSN industry with simple yet precise activation in nontoxic solvents, such as water. Notably, toxic and undesired polar aprotic solvents are generally used for the activation of membranes;^23,24^ however in this study, water is being reported for membrane activation.

Nanofiltration experiments were conducted to purify API from carcinogenic impurities. Results revealed that there was no API loss for the BioIPN^10^ membrane and that the loss was negligible for BioIPN^15^; however, there was a noticeable loss for BioIPN^20^ with an increasing number of diavolumes. The simulation results enabled us to determine the time and diavolumes required to reach the target impurity ratios (30, 10, and 5 ppm) based on the threshold of toxicological concern. Although the BioIPN^20^ structure only required two diavolumes to reach low impurity levels, the tightest membrane required substantially more solvent and time. The tradeoff between the amount of retained API and the amount of solvent required to reach optimal purity was therefore numerically verified. For the BioIPN^15^ membrane, which showed negligible API loss, the solvent consumption showed an approximately linear relationship with the number of diavolumes.

The measured data points (Figures 4h and 4i) for the BioIPN^15^ membrane were in good agreement with the model performance. The root-mean-square error (RMSE) values for impurity removal and API loss were 4.07% and 2.34%, respectively, whereas the impurity ratio and solvent consumption metrics had RMSE values of 3.85 ppm and 0.170 L g^–1^, respectively. We demonstrated that 5.1 diavolumes, with a negligible 0.56% API loss, were sufficient to reduce carcinogenic impurities to below the threshold level of toxicological concern.

## Conclusions

Herein, we fabricated a BioIPN membrane from natural materials (agarose and latex) using water as a solvent and without the addition of toxic cross-linking agents. The addition of latex to agarose increased the thermal and mechanical stabilities of the IPN membranes. Cryo-SEM analysis accurately revealed a swelling degree of ∼158% ± 1% for the presented BioIPN^0^, as well as the formation of structural microvoids after an activation time of 15 s in water. We demonstrated that IPN formation is an efficient strategy for controlling the swelling, polarity, and density of the membranes. In our polymer packing investigations, membrane densities gradually decrease as the latex concentration in the systems increases, thereby increasing the FFV.

The pore size and nanofiltration performance of the fabricated IPN membranes can be controlled by fine-tuning their chemical composition, processing conditions, and activation time in water. The MWCO values of the optimized membrane (BioIPN) gradually increased from 227 ± 27 to 623 ± 45 g mol^–1^ as the activation time in water increased from 10 s to 20 s. Moreover, the fabricated IPN membrane demonstrated long-term stability over 72 h of continuous crossflow nanofiltration at 20 bar. The rejection values of oleuropein and Methyl Orange were stable at ∼99.5% and ∼88.1%, respectively, with an average acetone flux of 53 L m^–2^ h^–1^.

The membranes were successfully used for API purification and removed a carcinogenic impurity below the threshold level of toxicological concern. Moreover, the IPN demonstrated high biodegradability over 14 days of enzymatic treatment, thereby ensuring the sustainable end of life of the membranes. Our method for sustainable and cost-effective fabrication of green free-standing IPN without the addition of toxic cross-linking agents provides an alternative to the current hazardous routes of membrane fabrication.

## Methods

### Materials

IPN were fabricated using SeaKem HE agarose (STDAG) derived from agar with a gelling temperature of 34.5–37.5 °C and Getahindus natural rubber latex concentrate of grade G-TEX LATZ. Prior to use, the low content of ammonia that was used to stabilize the natural rubber latex concentrate was removed from the concentrate via dialysis by employing a VWR regenerated cellulose membrane (MWCO = 1 kDa). The pH values of the dialysis medium were measured every day for 3 days until neutrality (pH 7) was achieved. The solid content of the natural rubber latex was quantified; the results are listed in Table S1. The solvents used in this study included ultrapure Milli-Q water, ethanol (99.7%, VWR Chemicals), and methanol (HPLC grade, VWR Chemicals). Arabian light crude oil was provided by Saudi Aramco. Diesel oil was purchased from a local fuel station in Thuwal, Saudi Arabia. Soybean oil (Alfa Aesar) was used as received. The enzymes used in the study were agarase (0.5 U μL^–1^, Thermo Fisher Scientific) and laccase from *Trametes versicolor* (≥0.5 U mg^–1^, Sigma–Aldrich). The chemicals used for the buffers in the enzymatic studies were Tris base, hydrochloric acid, and glacial acetic acid obtained from Fisher Scientific and sodium acetate purchased from VWR Chemicals. Novatexx 2471 polypropylene fibrous (PP) membrane was purchased from Freudenberg Filtration Technologies. All of the chemicals were used as received without additional purification.

### Membrane Fabrication

A 1.5 wt % aqueous solution of agarose was prepared by heating it to 100 °C under 300 rpm while stirring for 20 min. The solution was cooled to 50 °C, which is above the gelation temperature of agarose. Then, 10 wt % of latex was added for the fabrication of the BioIPN, and the system was stirred for 20 min. A 30 mL solution was then poured into a Petri dish (100 mm diameter) and kept at room temperature for 24 h. The benchmark membranes of pure agarose and pure latex were designated as Agarose and Latex, respectively. The membranes were subsequently immersed in a 10 L coagulation bath of ethanol for 24 h to facilitate water–ethanol exchange, followed by vacuum drying at 60 °C overnight. To investigate the effect of solvent exchange and solvent removal kinetics, the systems were also prepared without immersion in ethanol and with subsequent drying at 60 °C without vacuum (Table S2). Lastly, BioIPN^10^, BioIPN^15^, and BioIPN^20^ were fabricated by activating BioIPN^0^ in deionized water for a duration of 10, 15, and 20 s, respectively, in order to identify the effect that activation time had on membrane performance. The membrane designations are listed in Table 1. The material characterization specification can be found in Appendix A in the Supporting Information.

### Material Characterization

TGA was conducted using a Q5000 SA dynamic vapor sorption analyzer from TA Instruments at a heating rate of 10 °C min^–1^ up to 100 °C, followed by an isothermal hold at 100 °C for 30 min, and a subsequent 5 °C min^–1^ gradient up to 650 °C. Nitrogen was used as a protective gas for purging. DSC was performed using a DSC250 instrument (TA Instruments) with a nitrogen flow rate of 50 mL min^–1^ and a heating rate of 5 °C min^–1^ from −90 °C to 200 °C. This was followed by subsequent cooling at a rate of 5 °C min^–1^ from 200 °C to −90 °C. The DSC data processing was performed using TRIOS software from TA Instruments. The ATR-FTIR spectra of all samples were recorded using a Thermo Scientific Nicolet iS10 FTIR spectrometer. The spectra were obtained as an average of 64 scans at a resolution of 4 cm^–1^. Nano-FTIR spectroscopy (Neaspec GmbH) was conducted by using a laser centered at a wavelength of ∼1000 cm^–1^. In particular, 50-point FTIR spectra were collected along a 1000 nm scanning line, which resulted in a spatial resolution of 20 nm. A Pt/Ir-coated AFM tip with a frequency of 75 kHz was used. The membrane sample was attached to a silicon wafer by taping the edges of the membrane using silver tape. A standard TGQ1 reference sample was used to optimize the signal from the instrument. An area measuring 5 μm × 5 μm was selected for collecting surface topography data, and the nano-FTIR spectra were collected in the line-scan mode at a spatial resolution of 20 nm. A Nova Nano scanning electron microscope was used to investigate the morphology of the fabricated membranes. The samples were coated with a 5 nm layer of Pt using a sputtering system prior to the SEM measurement. WCA measurements were performed in triplicate on the membranes using a drop shape analyzer (KRÜSS Scientific, Model DSA100E) with the Young–Laplace method. The surface topographies of the membranes were characterized via tapping-mode AFM (Dimension Icon SPM, Bruker, Model RTESPA-300 probe). The tensile properties were obtained following the ASTM D882-02 standard test method, using a dynamic mechanical analyzer (TA Instruments Q800) with a loading rate of 1 N min^–1^ at room temperature. The nanoindentation technique was used to evaluate the mechanical hardness of the membranes by using a Micro Materials NanoTest Vantage instrument with a pyramidal diamond indenter at room temperature. Membrane samples of 1 cm^2^ size were attached to a silicon wafer using powerful adhesive. At least two indentations were performed per specimen, and the results were derived using the Berkovich beta factor with a manual polynomial general function. A molecular model and the FFV of the membranes were obtained using Materials Studio software (BIOVIA 2020) after determining the density of each membrane via Archimedes’ principle, using the liquid saturation method in *iso*-octane (ρ_25 °C_ = 0.68 g mL^–1^). The lattice parameter for all cubic cells presented by the model was set to 30 Å, and the simulation was performed at a standard ambient temperature. The simulation was performed by assigning the polymer distribution in a random manner, because it is technically impossible to assign a particular domain. Cryo-SEM was performed to further characterize the microstructure of BioIPN^0^ while mimicking the OSN conditions in which the IPN would be used. A 1 cm^2^ sample of BioIPN^0^ was activated for 15 s in ultrapure Milli-Q water and then immersed in acetone. BioIPN^0^ was later plunge-frozen in liquid nitrogen inside an electron microscopy–vacuum cryomanipulation (EM–VCM) system (Leica Microsystems) and manually freeze-fractured. The fractured sample was fixed in a freeze-fracture holder that was precooled inside the EM–VCM system and immediately transferred via a shuttle (Leica, Model VCT500) under cryogenic temperature into a freeze-fracture system (Leica, Model ACE900), where it was etched at −100 °C for 2 min and then finally was coated with a 4 nm layer of Pt. For the top surface analysis, the sample was placed in the holder so that the top surface was facing up; subsequently. The sample was transferred into the ACE900 freeze-fracture system, etched, and coated with a 4 nm layer of Pt. Both the cross-section and top surface were imaged inside the Helios G4 equipped with a Leica cryostage, which was precooled to below −140 °C. All cryo-SEM images were captured under cryogenic temperatures of less than −140 °C.

### Nanofiltration

The separation performance of the membranes was determined by using a crossflow nanofiltration system (Figure S12). To reduce the concentration polarization, a recirculation pump (Michael Smith Engineers, Ltd., U.K.) was used. All of the membranes were placed in cells of the nanofiltration system with an active area of 18 cm^2^, and they were then conditioned at 30 bar for 16 h in acetone as the retentate was recirculated at 1.2 L min^–1^. The solvent permeance and solute rejection were calculated according to eqs 1 and 2, respectively. Styrene dimer (236 g mol^–1^), estradiol (272.38 g mol^–1^), Methyl Orange (327.33 g mol^–1^), losartan (422.92 g mol^–1^), valsartan (435.52 g mol^–1^), oleuropein (540.51 g mol^–1^), acid fuchsin (585.54 g mol^–1^), roxithromycin (837.05 g mol^–1^), and rose bengal (1017.65 g mol^–1^) were used as standards for the nanofiltration. The concentrations of all of the other solutes in the feed stream were 10 μM.
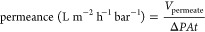
1
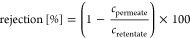
2where *V* is the volume of solvent permeating through a given membrane area *A* at time *t*, and *c*_permeate_ and *c*_retentate_ are the solute concentrations of the permeate and retentate, respectively. The MWCO was calculated from the rejection profiles at 90% rejection from two separate runs using independently prepared membranes.

### API Purification

Pharmaceutical purification was demonstrated via diafiltration using a procedure from our previous work.^25^ The membranes were conditioned in acetone for 16 h prior to use. The API was roxithromycin (837 g mol^–1^), and the carcinogenic impurity was 2-methoxyethoxymethyl chloride (125 g mol^–1^). Diafiltration is a constant-volume process in which the feed tank concentration (*c*), the retentate concentration (*c*_r_), and the permeate efflux concentration (*c*_p_) over time can be described with a system of ordinary differential equations (ODEs), as presented in eqs 3–5. In these equations, *P* is the solvent permeance, Δ*p* the transmembrane pressure drop, *A* the membrane area, *V* the volume of the solution, θ the ratio of the permeate and feed flow rates, and *R* the rejection of the compound.
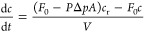
3
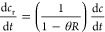
4
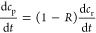
5

The solute concentrations were maintained at 1 g L^–1^ and 100 ppm in the crude API feed stream. The total volume of the diafiltration rig was kept constant at 500 mL, and the pressure was maintained at 20 bar. Because of the high feed flow rate, we approximated the retentate concentration using the feed concentration (*c*_r_ ≈ *c*), which allowed the model to be reduced to eqs 6 and 7. We used this approximation to solve the equations for API and impurities in a parallel manner:
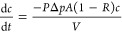
6
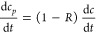
7

The parameter uncertainties were handled using a Monte Carlo approach with the assumption that the API rejection, the impurity rejection, and the solvent flux followed Gaussian distributions. Diafiltration simulations were performed with 1000 samples taken from the normal distributions as determined by the measured nominal means and standard deviations of the parameters. The trajectories of the 25^th^ and 75^th^ percentiles were highlighted and plotted as part of the data analysis. The ODE system was solved using Python3, using the odeint function of the sciPy package. Upon obtaining the concentration profiles over time, performance metrics were used to describe the efficiency of the process. The impurity ratio, API loss, impurity removal, and solvent consumption are defined in eqs 8–11, respectively, where *m* stands for mass and *V*_solvent_ is the total volume of solvent added to the system. By definition, *V*_solvent_ is also equal to the total amount of permeate drawn from the membrane.
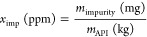
8
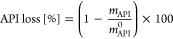
9
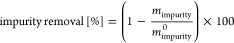
10
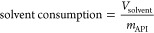
11

The number of diavolumes is defined as the ratio of the total amount of solvent and the system volume and therefore has a linear relationship with time:
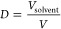
12

### Biodegradability

BioIPN^0^, agarose, and latex, together with a PP fibrous membrane control, were subjected to biodegradability testing. A dual-enzyme system was needed to degrade the IPN consisting of agarase (to degrade the agarose)^26^ and of Laccase (to degrade the latex).^27^ Samples of 1 cm^2^ size were treated in 10 mL of agarase at a concentration of 0.625 U mL^–1^ in 50 mM Tris–HCl buffer of pH 8.0 for 7 days. The biodegradation was carried out inside an IKA KS 4000 incubator shaker at 40 °C and 60 rpm. After 1 week, the remaining samples were washed with deionized water, dried, and subjected to the second enzyme treatment. The samples were placed in 10 mL of 1 U mL^–1^ Laccase in 10 mM acetate buffer at pH of 5. The Laccase treatment was performed at 37 °C at 60 rpm using the same incubator shaker for 7 days. Following the biodegradability test, the remaining membranes were collected on an NL 17 polyamide membrane filter having a pore size of 0.45 μm (Whatman, GE Healthcare Life Sciences) and thoroughly washed with deionized water. Then, the membranes were dried in an oven at 40 °C under a vacuum for a week. The weights of the dry samples before the start of the treatments and at the end of the second enzyme test were recorded, and the weight loss was calculated based on these values. The biodegradation experiments were carried out in duplicate using independently prepared BioIPN^0^ membranes.

### Computational Modeling

Models of latex and agarose were built by using the Build Polymer tool of the Materials Visualizer from the BIOVIA Materials Studio 2020 package. A fragment of the agarose structure in the form of a double helix from an experimental study^28^ was used as the initial building unit with one of the two chains of the double helix removed. The missing hydrogen atoms were adjusted by using the Materials Visualizer. The agarose–latex system was constructed by combining one latex chain and four agarose (single) chains in a cubic simulation box with 3500 water molecules using the Amorphous Cell module of the BIOVIA Materials Studio 2020 package and the COMPASS II force field.^29^ The density (ρ = 1 g cm^–3^) and temperature (*T* = 298 K) were used as target parameters. Geometry optimizations were executed by the Smart algorithm of the BIOVIA Materials Studio 2020 package with convergence criteria of 0.001 kcal mol^–1^ for the energy and 0.5 kcal mol^–1^ Å^–1^ for the force, no external pressure, and a number of optimization steps limited to 500. The unit cell parameter of 48.79 Å (as determined in the construction procedure) was maintained in the geometry optimization. The noncovalent interactions were analyzed by the NCIPLOT program with the charge density approximated as sum of the atomic charge densities.^30^ An electron density cutoff of 0.06 a.u. was sufficient for describing the noncovalent interactions. MD simulations were carried out with the Forcite program of the BIOVIA Materials Studio 2020 package. Using a canonical ensemble and the COMPASS II force field, they were run for 9 ns with a time step of 1 fs. The temperature was set to 298 K, controlled by an Andersen thermostat. The nonbonding Coulombic and van der Waals interactions (cutoff distance of 12 Å) were evaluated by the Ewald summation method with an accuracy of 0.001 kcal mol^–1^. Binding energies were calculated by single-point calculations using the Gaussian 09 package with M06-2X functional, the 6-31+G** basis set, and the water described by a polarizable continuum model.

## Supporting Information Available

Details on latex solid content determination, membrane designations, hydrogel formation, solvent stability analysis, thermomechanical and chemical analysis, biodegradation, membrane nanofiltration, pore-size calculations, and molecular simulations (PDF)
