# Supplementary material for: Biobased Interpenetrating Polymer Network Membranes for Sustainable Molecular Sieving
Source: ACS Nano. 2024 Feb 20;18(10):7433–43. doi: 10.1021/acsnano.3c10827 (PMC10938919; doi:10.1021/acsnano.3c10827)
Supplement: Supplementary file 1 — nn3c10827_si_001.pdf [file nn3c10827_si_001.pdf]

Supporting Information

Biobased Interpenetrating Polymer Network Membranes for Sustainable Molecular Sieving

Joyce Cavalcante[1,2], Diana G. Oldal[1,3], Maxim V. Peskov[2], Aron K. Beke[1,3], Rifan Hardian[1], Udo Schwingenschlogl[2], Gyorgy Szekely[1,2,3*]

[1] Advanced Membranes and Porous Materials Center, Physical Science and Engineering Division (PSE), King Abdullah University of Science and Technology (KAUST), Thuwal 23955-6900, Saudi Arabia

[2] Materials Science and Engineering Program, Physical Science and Engineering Division (PSE), King Abdullah University of Science and Technology (KAUST), Thuwal, 23955-6900, Saudi Arabia

[3] Chemical Engineering Program, Physical Science and Engineering Division (PSE), King Abdullah University of Science and Technology (KAUST), Thuwal, 23955-6900, Saudi Arabia

[*] Corresponding author: +966128082769, gyorgy.szekely@kaust.edu.sa, www.szekelygroup.com

Table of contents

List of figures S2
List of tables S2
1. Latex solid content (LSC) S3
2. Membrane designations S3
3. Hydrogel formation S4
3. Solvent resistance analysis S5
4. Thermal, mechanical, and chemical analysis S8
5. Biodegradation S11
7. Membrane nanofiltration S15
8. Pore-size calculations S16
9. Molecular simulation S25
References S32

List of figures

Figure S1: Hydrogel formation after physical crosslinking of agarose S4
Figure S2: Correlation between the water contact angle measurements and swelling degree S7
Figure S3: Swelling as a function of solvent polarity.. S7
Figure S4: TGA to show the increase in thermal stability with the gradual addition of latex. ... S8
Figure S5: Nanoindentation profiles and hardness values S9
Figure S6: Characterization of the fabricated membranes... S11
Figure S7: Membranes before the biodegradability tests. S12
Figure S8: (a–d) Scanning electron microscopy (SEM) cross-sections, (e–h) SEM top surfaces. The water contact angle of each membrane is presented as an inset in each subfigure of Figure 3e–h. (i–l) AFM three-dimensional projections for Agarose, BioIPN$_{05}$, BioIPN^{0}, and BioIPN$_{15}$ ... S13
Figure S9: SEM cross-section indicating membrane thickness of (a) Agarose, (b) BioIPN$_{05}$, (c) BioIPN^{0}, and (d) BioIPN$_{15}$.. S13
Figure S10: Deleterious defects found on BioIPN$_{15}$ at both (a and b) microscale and (c) macroscale due to chemical incompatibilities in the IPN system. S14
Figure S11: Cryo-SEM top surface analysis for BioIPN^{0} at different magnifications: (a) 1000×, (b) 2500×, and (c) 5000×. S14
Figure S12: Schematic of the multistage cross-flow nanofiltration apparatus used for membrane testing. Note that flat sheet membranes were used in the experiments. S15
Figure S13: Rejection profiles of BioIPN^{0} for different activation times in water S15
Figure S14: Evaluation of the standard deviation for rejection and flux as a function of time . S16
Figure S15: Nanofiltration performance of the IPN membranes.. S20
Figure S16: Pure solvent flux profiles for BioIPN^{15} at 20 bar.. S21
Figure S17: API loss, impurity removal, impurity ratio, and solvent consumption S22
Figure S18: Molecular simulation of the polymer packing S26
Figure S19: Schematic drawing of BioIPN^{0}'s: (a) predicted 3D swelling in virtual space according to the 2D swelling degree found via (b) SEM and (c) cryo-SEM. S28
Figure S20: Molecular simulation of the (a, b) polymer packing and (c, d) FFV in BioIPN^{0} before and after 15 s activation in water. S29

List of tables

Table S1: Latex solid content (LSC) quantification S3
Table S2: Membrane designations.. S4
Table S3: Dissolution tests were conducted for up to 24 h in 11 different solvents.................... S6
Table S4. Thermal parameters of the investigated membrane systems. S8
Table S5: Mechanical features of the investigated membrane systems...................................... S9
Table S6. Physical properties of acetone S16
Table S7: Pure solvent flux determination at 20 bar for different solubility parameters........... S24
Table S8: Permeance and MWCO of the BioIPN and other membranes S32

1. Latex solid content (LSC)

The latex solid content was determined according to the following equation:

$$LSC = \frac{\text{solid weight (g)}}{\text{humid weight (g)}} * 100$$

Table S1: Latex solid content (LSC) quantification, where LA, LB, and LC are three different latex samples. Samples were dried under an infrared lamp overnight (50 °C).

	Humid weight (g)	Solid weight (g)	LSC (%)
LA	1.00	0.48	48.74
LB	1.00	0.48	48.26
LC	1.00	0.48	48.73
Average LSC (%)	48.58 ± 0.27		

2. Membrane designations

A 1.5 wt% aqueous solution of agarose was prepared by heating to 100 °C under stirring at 300 rpm for 20 min. The solution was cooled to 50 °C—which is above the gelation temperature of agarose—and then latex was added in the amount of 5, 10, and 15 wt% to $BioIPN^{05}$, $BioIPN^{0}$, and $BioIPN^{15}$ respectively, and the system was stirred for 20 min. A 30 mL solution was then poured into a petri dish (10 cm in diameter) and kept at room temperature for 24 h. The benchmark membranes of pure agarose and pure latex were designated as Agarose and Latex, respectively. The systems were subsequently immersed into a 10-L coagulation bath of ethanol for 24 h to facilitate water-ethanol exchange, followed by vacuum drying at 60 °C overnight. To investigate the effect of solvent exchange, $BioIPN_{NSE}$ was prepared as $BioIPN^{0}$ but without the immersion in ethanol. The effect of the solvent removal kinetics upon drying was evaluated by preparing $BioIPN_{NVD}$ similarly to the preparation of $BioIPN^{0}$, with subsequent drying at 60 °C without vacuum. $BioIPN_{NSEVD}$ was prepared without solvent exchange and without vacuum drying. Lastly, $BioIPN^{10}$, $BioIPN^{15}$, and $BioIPN^{20}$ were fabricated by activating $BioIPN^{0}$ in deionized water for

10, 15, and 20 s, respectively, in order to identify the effect that activation time had on membrane performance. The membrane designations are shown in Table S2.

Table S2: Membrane designations according to the conditions of fabrication and composition. Ethanol was used in the coagulation bath for solvent exchange, and a vacuum of 30 mbar was used for drying. *Benchmark membranes.

Membrane	$C_{agarose}$ (wt%)	C_{latex} (wt%)	Solvent exchange	Vacuum drying	IPN	Activation (s)
Agarose	100	0	Yes	Yes	No*	0
BioIPN$_{05}$	95	5	Yes	Yes	Yes	0
BioIPN$^{\mathbf{0}}$	90	10	Yes	Yes	Yes	0
BioIPN$_{15}$	85	15	Yes	Yes	Yes	0
Latex	0	100	Yes	Yes	No*	0
BioIPN$_{NSE}$	90	10	No	Yes	Yes	0
BioIPN$_{NVD}$	90	10	Yes	No	Yes	0
BioIPN$_{NSEVD}$	90	10	No	No	Yes	0
BioIPN^{10}	90	10	Yes	Yes	Yes	10
BioIPN^{15}	90	10	Yes	Yes	Yes	15
BioIPN^{20}	90	10	Yes	Yes	Yes	20

3. Hydrogel formation

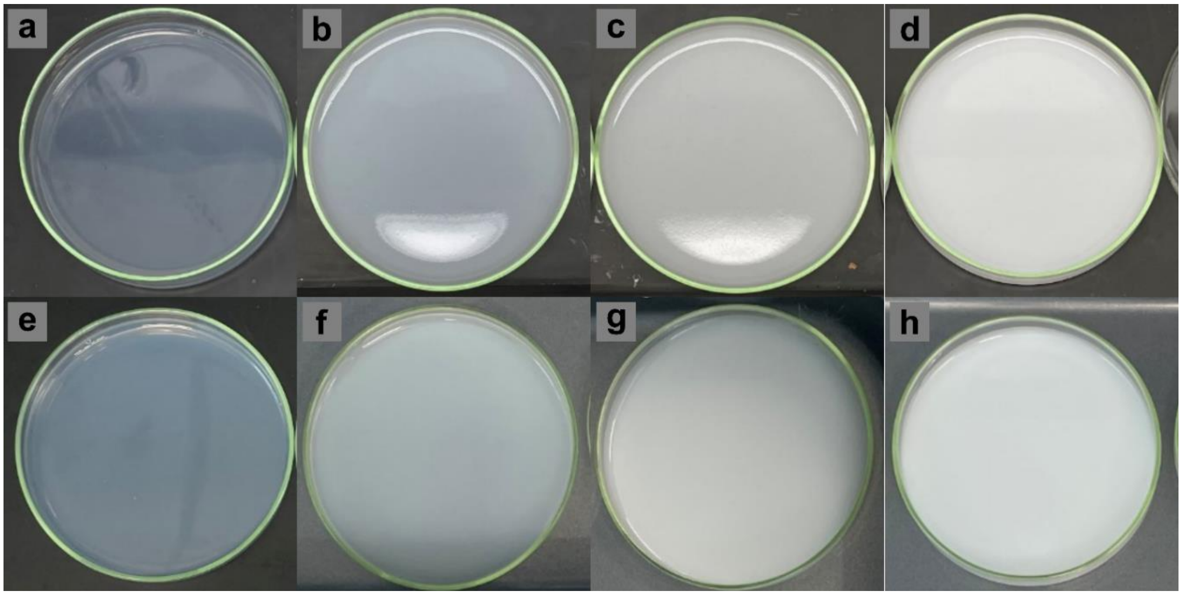

Figure S1: Hydrogel formation after physical crosslinking of agarose at ambient temperature. Semi IPN hydrogels for (a) Agarose, (b) BioIPN$_{05}$, (c) BioIPN^{0}, (d) BioIPN$_{15}$, and IPN hydrogels after time-driven covalent crosslinking of latex overnight for (e) Agarose, (f) BioIPN$_{05}$, (g) BioIPN^{0}, and (h) BioIPN$_{15}$. Displayed Petri dishes have a 10 cm diameter.

3. Solvent resistance analysis

Membranes were fabricated with commercial agarose and natural rubber latex. After 24 h, a tiny degree of swelling was noted in all cases. Mild swelling was noticed in water (the solvent that was used to prepare the dope solution). The membranes immersed in dimethylformamide (DMF) were the most swollen. The presence of latex increased the swelling of membranes immersed in DMF. The membrane with 100% agarose dissolved completely in dimethyl sulfoxide (DMSO), whereas the latex membranes were only partially swollen. The greater the amount of latex added, the more stable the membranes seemed to be in DMSO (while still in the medium swelling category).

The degree of swelling (swelling degree) was determined according to the following equations for weight and thickness, respectively:

$$Swelling\ degree\ (\%) = \frac{\mathrm{Ww\ (g) - Wd\ (g)}}{\mathrm{Wd\ (g)}} * 100, \qquad \text{Eq. S1}$$

$$Swelling\ degree\ (\%) = \frac{\mathrm{Tw\ (g) - Td\ (g)}}{\mathrm{Td\ (g)}} * 100, \qquad \text{Eq. S2}$$

where Ww represents the weight of the wet sample, Wd represents the weight of the dried sample, Tw represents the thickness of the wet sample, and Td represents the thickness of the dried sample.

Table S3: Dissolution tests were conducted for up to 24 h in 11 different solvents.

	H_2O	Acetone	Acetonitrile	Ethanol	Ethyl acetate	Methanol	DMF	DMAc	DMSO	Chloroform	THF
**Agarose**											
1 min	✖ (green)	✖ (green)	✖ (green)	✖ (green)	✖ (green)	✖ (green)	✖ (green)	✖ (green)	✖ (green)	✖ (green)	✖ (green)
1 h	✖ (blue)	✖ (green)	✖ (green)	✖ (green)	✖ (green)	✖ (green)	✖ (yellow)	✖ (green)	✖ (yellow)	✖ (green)	✖ (green)
2 h	✖ (blue)	✖ (green)	✖ (green)	✖ (green)	✖ (green)	✖ (green)	✖ (yellow)	✖ (green)	✖ (yellow)	✖ (green)	✖ (green)
24 h	✖ (blue)	✖ (green)	✖ (green)	✖ (green)	✖ (green)	✖ (green)	✖ (yellow)	✖ (green)	● (red)	✖ (green)	✖ (green)
**$BioIPN_{05}$**											
1 min	✖ (green)	✖ (green)	✖ (green)	✖ (green)	✖ (green)	✖ (green)	✖ (green)	✖ (green)	✖ (green)	✖ (green)	✖ (green)
1 h	✖ (blue)	✖ (green)	✖ (green)	✖ (green)	✖ (green)	✖ (green)	✖ (yellow)	✖ (green)	✖ (yellow)	✖ (green)	✖ (green)
2 h	✖ (blue)	✖ (green)	✖ (green)	✖ (green)	✖ (green)	✖ (green)	✖ (yellow)	✖ (green)	✖ (yellow)	✖ (green)	✖ (green)
24 h	✖ (blue)	✖ (green)	✖ (green)	✖ (green)	✖ (green)	✖ (green)	✖ (red)	✖ (green)	✖ (yellow)	✖ (green)	✖ (green)
**$BioIPN^0$**											
1 min	✖ (green)	✖ (green)	✖ (green)	✖ (green)	✖ (green)	✖ (green)	✖ (green)	✖ (green)	✖ (green)	✖ (green)	✖ (green)
1 h	✖ (blue)	✖ (green)	✖ (green)	✖ (green)	✖ (green)	✖ (green)	✖ (yellow)	✖ (green)	✖ (green)	✖ (green)	✖ (green)
2 h	✖ (blue)	✖ (green)	✖ (green)	✖ (green)	✖ (green)	✖ (green)	✖ (yellow)	✖ (green)	✖ (green)	✖ (green)	✖ (green)
24 h	✖ (blue)	✖ (green)	✖ (green)	✖ (green)	✖ (green)	✖ (green)	✖ (red)	✖ (green)	✖ (yellow)	✖ (green)	✖ (green)
**$BioIPN_{15}$**											
1 min	✖ (green)	✖ (green)	✖ (green)	✖ (green)	✖ (green)	✖ (green)	✖ (green)	✖ (green)	✖ (green)	✖ (green)	✖ (green)
1 h	✖ (blue)	✖ (green)	✖ (green)	✖ (green)	✖ (green)	✖ (green)	✖ (yellow)	✖ (blue)	✖ (yellow)	✖ (green)	✖ (green)
2 h	✖ (blue)	✖ (green)	✖ (green)	✖ (green)	✖ (green)	✖ (green)	✖ (yellow)	✖ (blue)	✖ (yellow)	✖ (green)	✖ (green)
24 h	✖ (blue)	✖ (green)	✖ (green)	✖ (green)	✖ (green)	✖ (green)	✖ (red)	✖ (blue)	✖ (yellow)	✖ (green)	✖ (green)

✖ (green) Does not dissolve or swell
✖ (blue) Mild swelling
✖ (yellow) Medium swelling
✖ (red) Strong swelling
● (blue) Partially dissolves
● (red) Completely dissolves

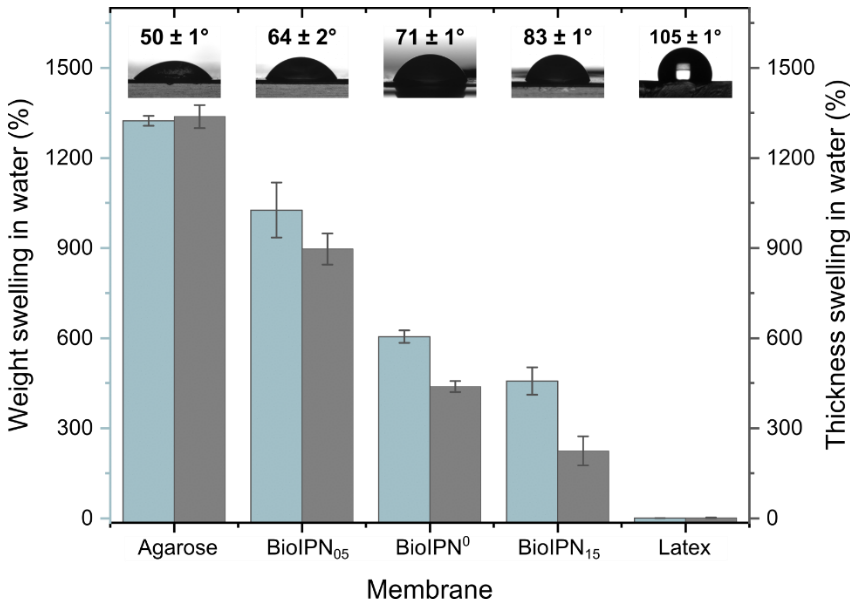

Figure S2: Correlation between the water contact angle measurements and swelling degree after 24 h of immersion in water. The weight and thickness were evaluated.

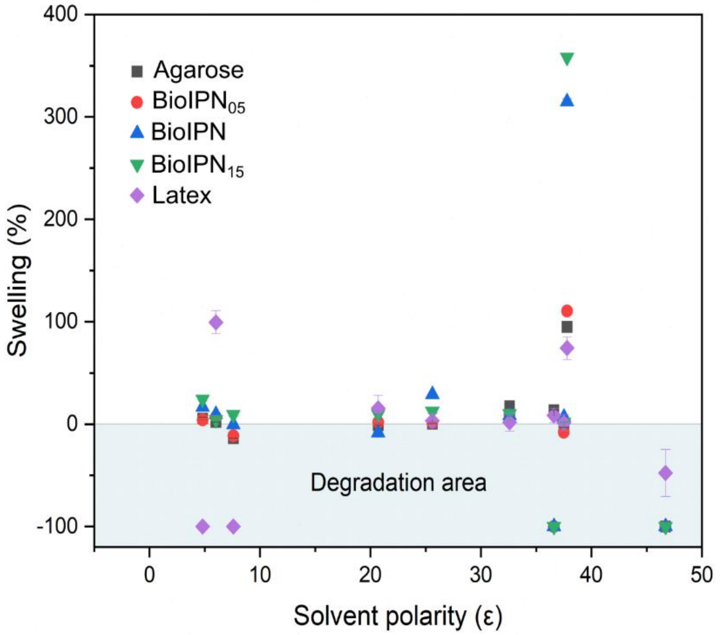

Figure S3: Swelling as a function of solvent polarity.

4. Thermal, mechanical, and chemical analysis

Table S4. Thermal parameters of the investigated membrane systems.

Membrane	T_g (°C)	Enthalpy of glass transition (ΔH_m, J g^{-1})
Agarose	95.60	634.55
$BioIPN_{05}$	106.06	590.11
$BioIPN^0$	105.67	577.07
$BioIPN_{15}$	105.97	533.00
Latex	–67.07	1.68

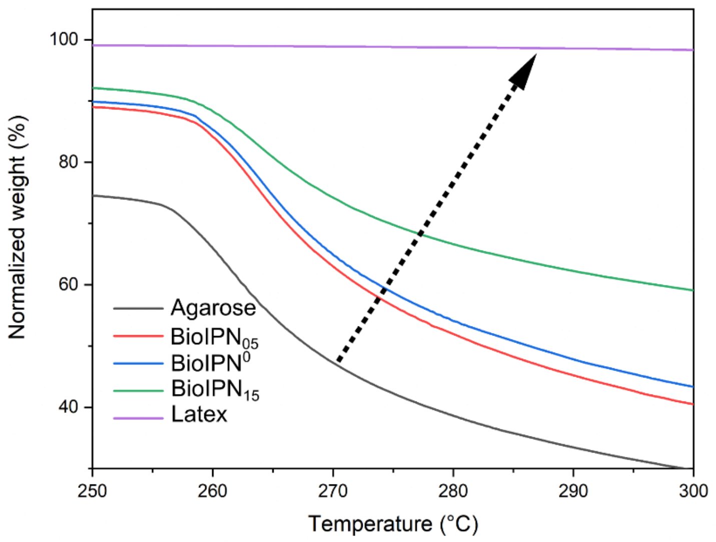

Figure S4: TGA to show the increase in thermal stability with the gradual addition of latex.

Latex exhibited a maximum elongation of 370.53 ± 59.57% (Table S5). This substantially large value (>200%) corroborates the classification of Latex as an elastomer.[1] Hence, under the presented processing conditions, the natural rubber latex presents a low density of crosslinking bonds confirmed by its elastomeric behavior. As predicted, the time-driven covalent crosslinking was confirmed by mechanical analysis. The crosslinking of natural rubber latex was first found to occur spontaneously under a neutral pH within 8–14 h,[2] where simple exposure to the ambient

conditions of pressure and temperature led to an oxidative crosslinking reaction on polyisoprene chains by microorganisms during coagulation.[3]

Table S5: Mechanical features of the investigated membrane systems.

	**Tenacity (MJ m^{-3})**	**Young's modulus (MPa)**	**Maximum tensile stress (MPa)**	**Maximum elongation (%)**
Agarose	74.38 ± 0.89	3.00 ± 0.35	37.44 ± 3.41	4.28 ± 1.27
BioIPN$_{05}$	48.73 ± 5.06	0.98 ± 0.31	24.34 ± 0.23	4.06 ± 0.08
BioIPN^{0}	217.23 ± 7.17	8.57 ± 0.25	47.30 ± 0.56	6.79 ± 0.03
BioIPN$_{15}$	170.99 ± 8.59	0.33 ± 0.11	35.47 ± 2.73	10.12 ± 0.83
Latex	112.18 ± 11.17	0.006 ± 0.02	0.39 ± 0.10	370.53 ± 59.57

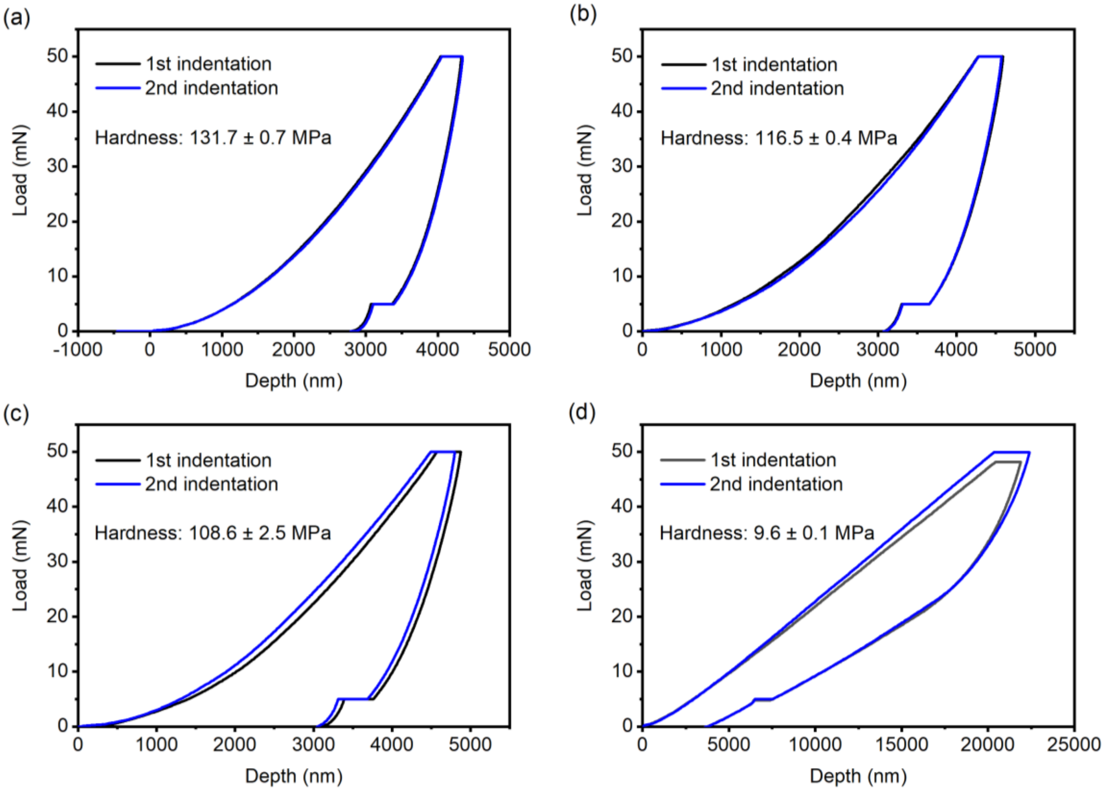

Figure S5: Nanoindentation profiles and hardness values for (a) Agarose, (b) BioIPN$_{05}$, (c) BioIPN^{0}, and (d) BioIPN$_{15}$.

The addition of natural rubber latex to BioIPN gradually reduces its hydrophilicity (Figure S2), which is indicated by the amount of moisture loss in stages i and ii in Figure S6a. This presents

an opportunity to control the thermal properties of the membrane. The thermal behavior of $BioIPN^0$ displayed three different stages (Figure S6a). The first stage (i) is related to the loss of moisture (until 100 °C), followed by a constant decay under isothermal conditions (stage ii) to completely remove the moisture at 100 °C. The plateau until approximately 300 °C represents a region of thermal stability in the investigated systems. The third stage is attributed to the thermal degradation of the covalent bonds of the polymers. The hydrophilic agarose pristine system (Agarose) displays the lowest thermal stability by presenting intense weight loss in stages ii and ii. Owing to the hydrophobic nature of latex (Figure S2), stages (i) and (ii) were virtually imperceptible, and only one decomposition step (iii) was observed after 300 °C. Notably, the gradual addition of latex caused an increase in thermal stability, which is indicated by the progressive shift in the curves in the direction of the upper right side of the panel (Figure S4). The TGA results for the pristine membranes Agarose and Latex are in accordance with previously reported data.[1,2] The DSC thermograms (Figure S6b) of the agarose-based membranes exhibited broad endothermic peaks ranging from 25 °C to 170 °C. These peaks represent the glass transition temperature (T_g) range of the investigated membrane systems during heating.[4] Additionally, the DSC curve of Latex was also investigated and revealed that T_g for the pure latex system is approximately −67 °C. The tenacity and Young's modulus of the membranes were evaluated to assess their mechanical properties (Figure S6c). Tenacity represents the amount of mechanical energy (MJ m^{-3}) absorbed by the material before catastrophic failure, and it is typically calculated by integrating the area below the strain vs. stress curves. Additional values for the maximum tensile stress and maximum elongation are presented in Table S5.

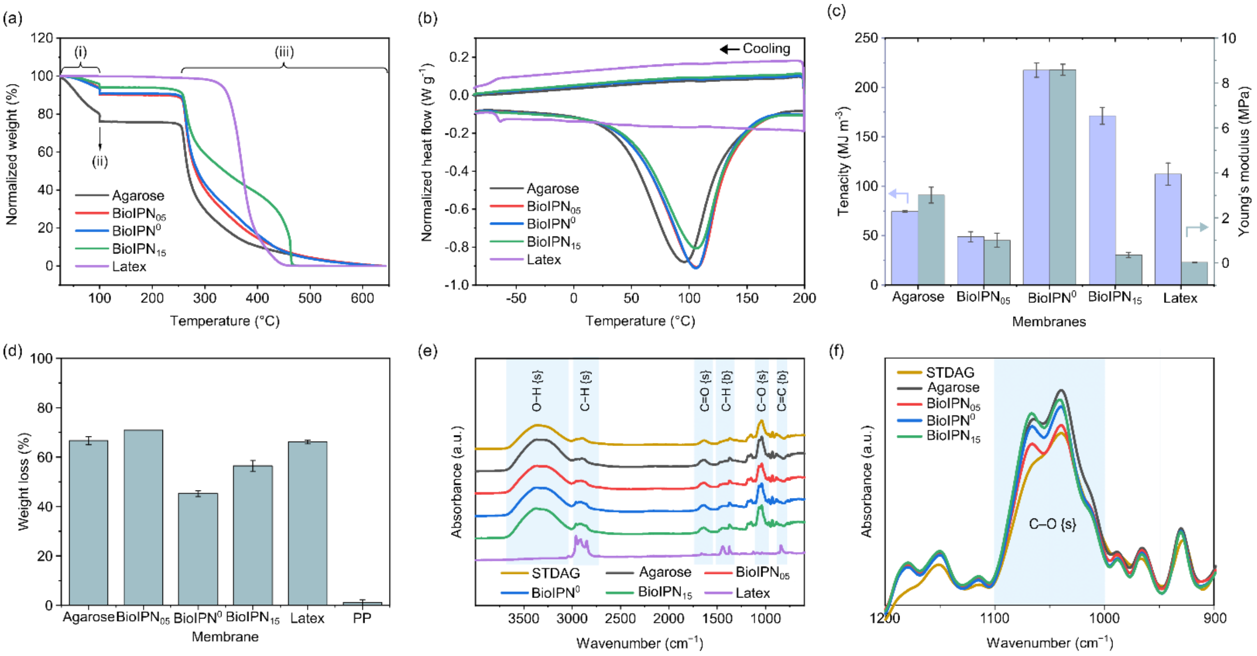

Figure S6: (a) TGA and (b) DSC spectra of the membranes fabricated in this study. (c) Tensile properties obtained via mechanical characterization of the investigated membranes. (d) Biodegradation analysis of membrane systems considering weight loss due to enzymatic treatment. (e) Chemical characterization via FTIR of the fabricated membranes. The {s} and {b} designations correspond to the peaks caused by the stretching and bending modes, respectively. (f) FTIR zoom in alcoholic C–O {s} region for detailed chemical characterization.

5. Biodegradation

The biodegradable nature of the membranes was evaluated using naturally occurring enzymes (agarase and laccase). A polypropylene (PP) fibrous membrane was used as a control, as it did not show any degradation during the total testing period of two weeks. All membranes exhibited excellent biodegradability upon enzymatic treatment (Figure S7). Agarose and Latex, the two non-IPN systems exhibited similar weight loss during the biodegradability tests. These results were expected, because both contain 100% agarose or latex, and they were subjected to both enzymes for an equal period of time.

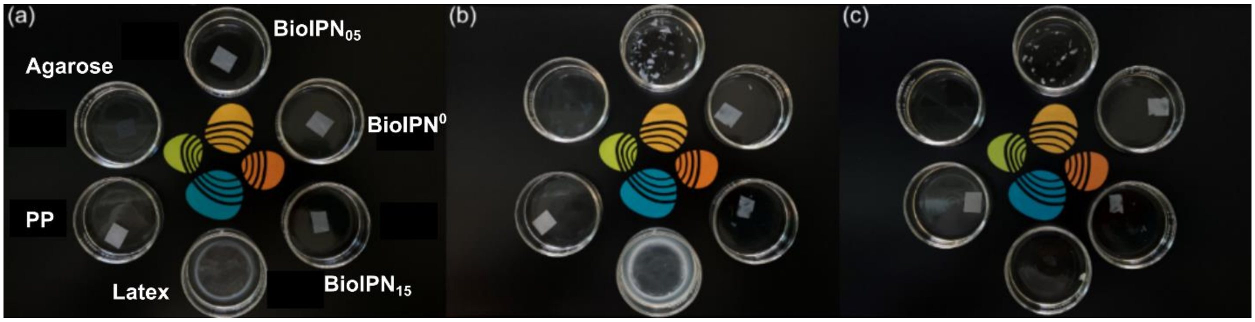

Figure S7: Membranes before the biodegradability tests (a); after the agarase treatment (b); and following the laccase treatment (c).

6. Morphological features

Lumps appeared on the surface of the membranes with increasing latex content in the IPN (Figure S8e–h). Moreover, the surface of the IPN membranes became more textured than that of the pristine agarose membrane. Atomic force microscopy analysis (Figure S8i–l) revealed an increase in the topographical texture with increasing latex content. The Ra increased by 98% with a 15 wt% addition of latex, which varied from 88.90 nm in Agarose to 176.55 nm in $BioIPN_{15}$. The hydrophobicity of the membranes increased with increasing latex content (Figure S8e–h); this can be attributed to the synergistic effects of the hydrophobic character of latex and the rougher surface of the membranes. The water contact angle measurements corroborated the swelling degree of the membranes in water, which was determined using both thickness and weight measurements (Figure S2). The increase in membrane wettability ($BioIPN_{15} \rightarrow$ Agarose) led to the higher swelling degree of Agarose compared with $BioIPN_{15}$. Thus, the addition of latex was also proven to be an efficient strategy for controlling swelling as well as superficial polarity and liquid–solid interactions. The water contact angle measurement results were in line with the TGA results related to the amount of moisture loss from the membrane surface, as explained earlier (Figure 2a).

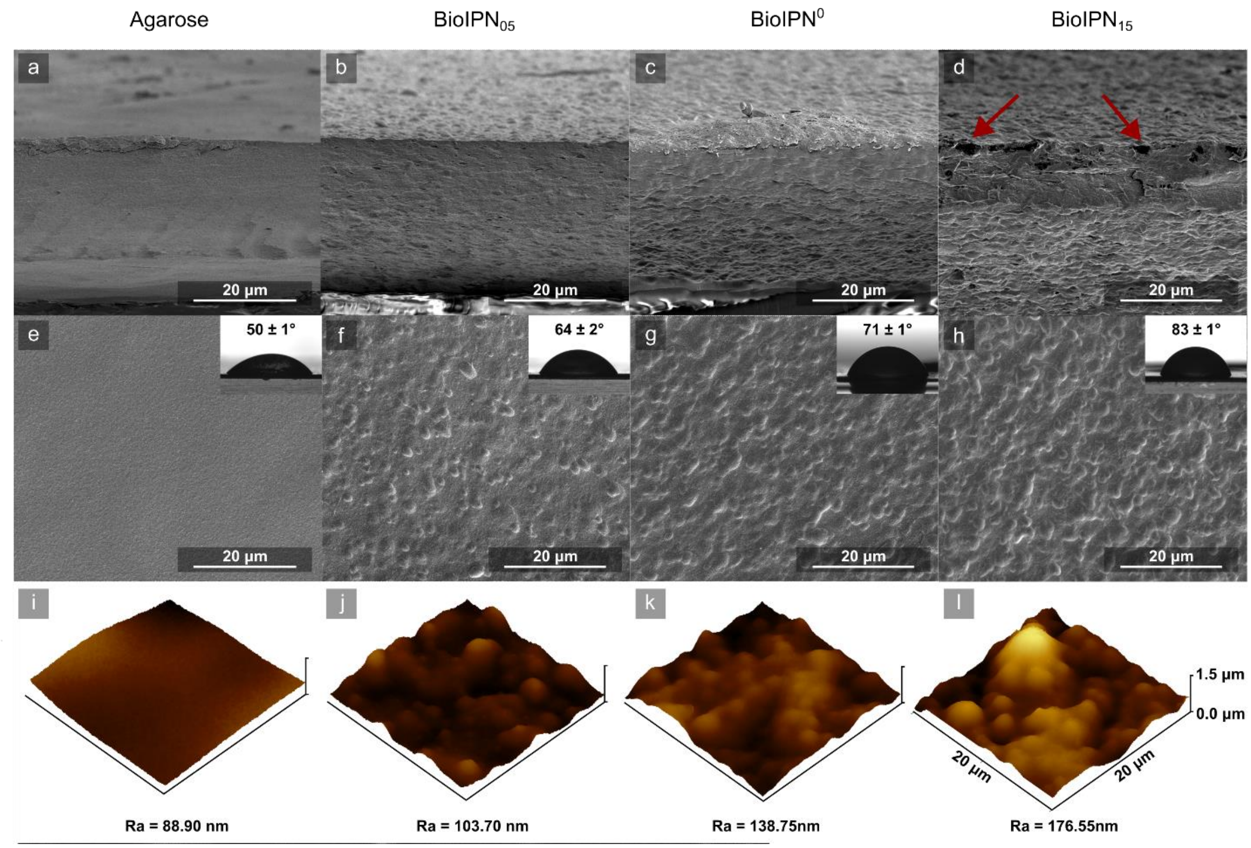

Figure S8: (a–d) Scanning electron microscopy (SEM) cross-sections, (e–h) SEM top surfaces. The water contact angle of each membrane is presented as an inset in each subfigure of Figure 3e–h. (i–l) AFM three-dimensional projections for Agarose, $BioIPN_{05}$, $BioIPN^{0}$, and $BioIPN_{15}$. All the AFM images were uniformly scaled with a fixed height bar (from 0 to 1.5 µm).

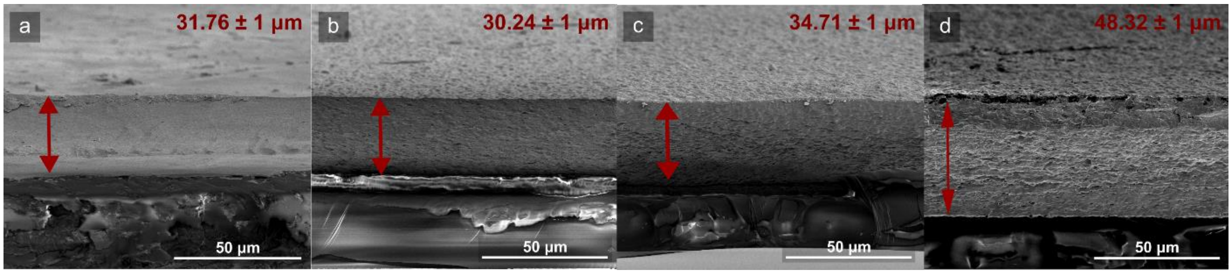

Figure S9: SEM cross-section indicating membrane thickness of (a) Agarose, (b) $BioIPN_{05}$, (c) $BioIPN^{0}$, and (d) $BioIPN_{15}$.

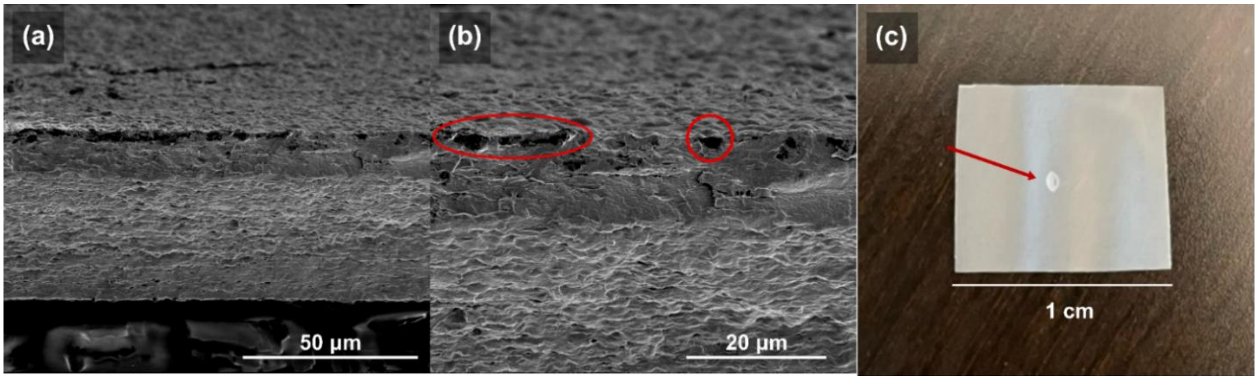

Figure S10: Deleterious defects found on $BioIPN_{15}$ at both (a and b) microscale and (c) macroscale due to chemical incompatibilities in the IPN system.

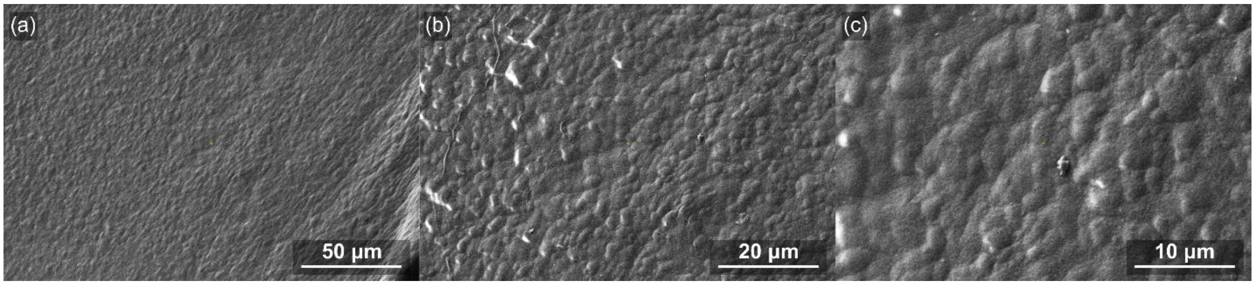

Figure S11: Cryo-SEM top surface analysis for $BioIPN^{0}$ at different magnifications: (a) 1000×, (b) 2500×, and (c) 5000×.

$BioIPN^{0}$ was selected for further nanofiltration testing due to its favorable mechanical properties, which include tenacity, Young's modulus, and maximum tensile stress (Figure 2d), and Table S5). Moreover, as density and swelling are known to be limiting factors of OSN performance,[5] $BioIPN^{0}$ was selected because it exhibited reasonable values for swelling, fractional free volume (FFV), and density compared with the other investigated systems (Table S2). Notably, $BioIPN^{15}$ was not selected for nanofiltration analysis owing to the presence of defects at both the microscale (microvoids were spotted via SEM on the top dense layer of the IPN) and macroscale (non-homogeneous mixing of latex during processing caused premature gelation to occur in specific spots, leading to phase separation due to the formation of evident agglomerates). The details for the defects are presented in Figure S10. The relatively low value of hardness (9.6 ± 0.1

MPa) obtained via nanoindentation (Figure S5) also contributed to the disqualification of $BioIPN_{15}$ from further analysis.

7. Membrane nanofiltration

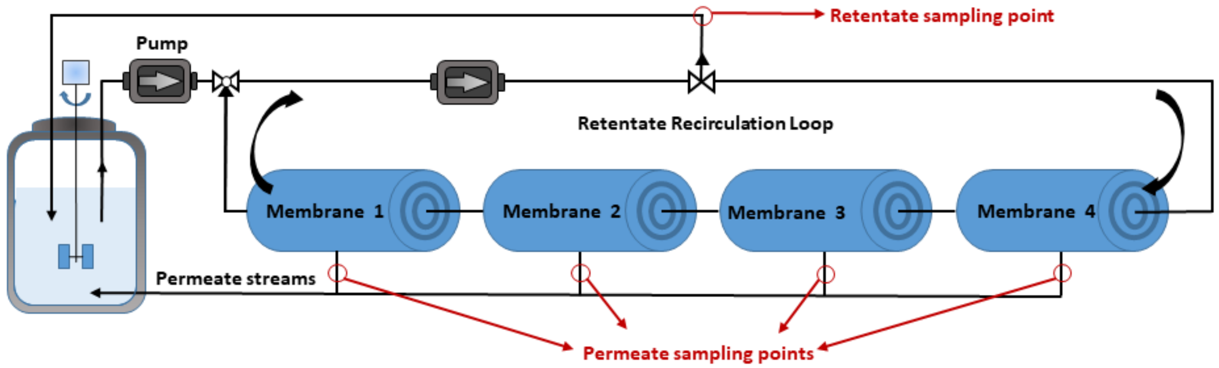

Figure S12: Schematic of the multistage cross-flow nanofiltration apparatus used for membrane testing. Note that flat sheet membranes were used in the experiments.

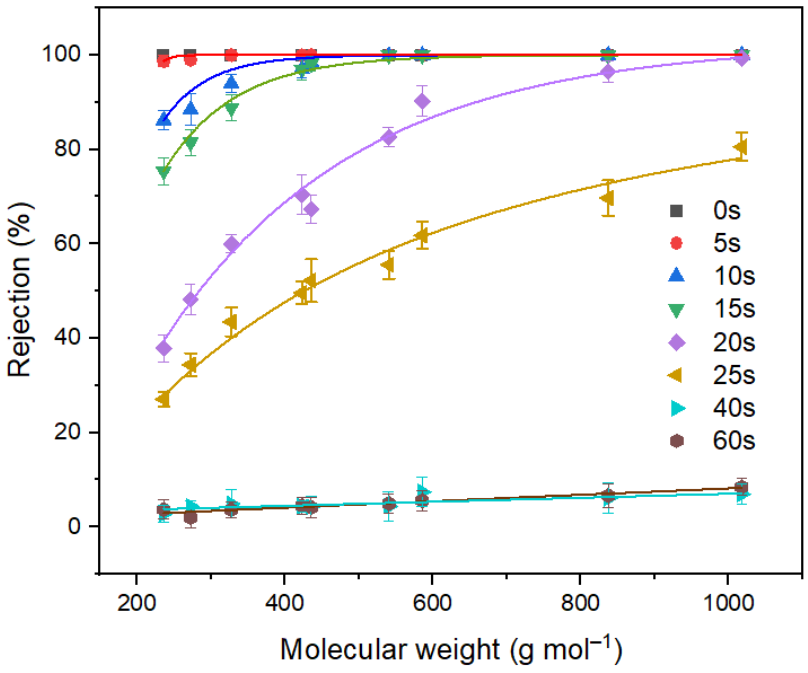

Figure S13: Rejection profiles of $BioIPN^0$ for different activation times in water (0, 5, 10, 15, 20, 25, 40, and 60 s).

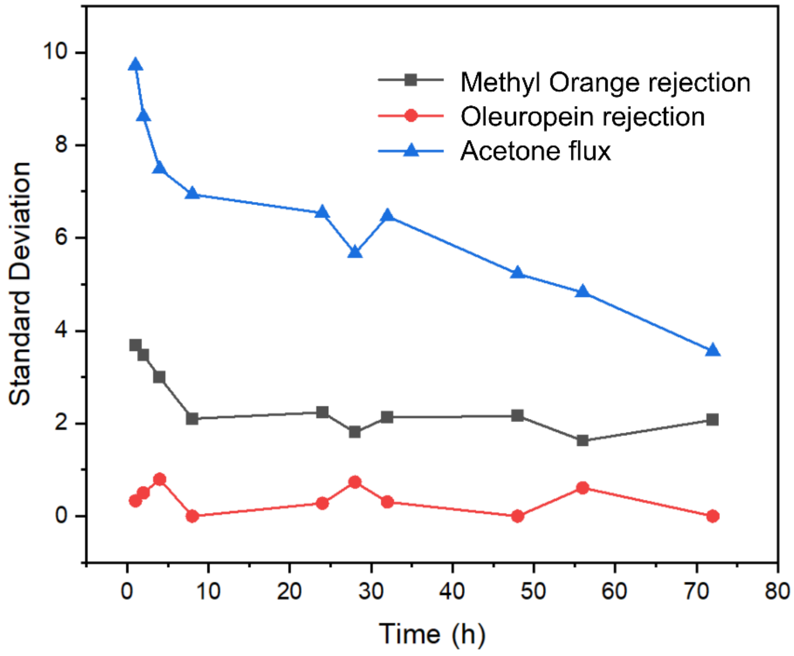

Figure S14: Evaluation of the standard deviation for rejection and flux as a function of time for BioIPN[15].

8. Pore-size calculations

Table S6. Physical properties of acetone.[6]

Solvent	M_w[a] (Da)	d_m[b] (nm)	η[c] (mPa s)	V_m[d] (cm^3 mol^{-1})	ρ[e] (g ml^{-1})	δ_d[f] (MPa$^{0.5}$)	δ_P[g] (MPa$^{0.5}$)	δ_h[h] (MPa$^{0.5}$)	δ_t[i] (MPa$^{0.5}$)
Acetone	58.1	0.618	0.3	74.08	0.784	15.5	10.4	7	19.94

[a] Molar mass; [b] diameter; [c] dynamic viscosity; [d] molar volume; [e] density; [f,g,h,i] Hansen parameters (dispersion, polar, hydrogen bonding, and total, respectively).

As suggested by Livingston et al.,[7] the permeance of a solvent can be correlated to its physical properties. The diameter of acetone was calculated as follows:

$$d_{\mathrm{m}} = 2 \cdot \left(\frac{3V_m}{4\pi N_A}\right)^{\frac{1}{3}}, \qquad \text{Eq. S3}$$

where V_m represents the molar volume obtained from the solvent density and N_A represents Avogadro's number. The Hagen–Poiseuille equation defines the volumetric flux (J_v) through a membrane comprised of uniform capillaries:

$$J_{v,i} = \frac{r_i^2 \Delta P \varepsilon}{8\mu_0 l}, \qquad \text{Eq. S4}$$

where ε represents the porosity, ΔP represents the transmembrane pressure, l represents the capillary length, μ_0 represents the solvent bulk viscosity, and r_i represents the capillary radius. Next, using the pore flow rate ($Q_{p,i}$), the flow through a pore of radius r_i was calculated as follows:

$$Q_{p,i} = \frac{\pi r_i^4 \Delta P}{8\mu_0 l}. \qquad \text{Eq. S5}$$

The overall solute rejection was calculated using the following equation:

$$R_{ij} = 1 - \frac{\Phi_{ij} K_{c,ij}}{1 - (1 - \Phi_{ij} K_{c,ij})(\exp(-P_{e,ij}))}, \qquad \text{Eq. S6}$$

where Φ_{ij} represents the partition coefficient and λ_{ij} represents the ratio between the solute radius $r_{s,j}$ (the subindex j indicates the solute) and the pore radius r_i (the subindex i indicates the pore-size class in the discretization method). Φ_{ij} and λ_{ij} can be calculated as follows:

$$\Phi_{ij} = (1 - \lambda_{ij})^2; \qquad \text{Eq. S7}$$

$$\lambda_{ij} = \frac{r_{s,j}}{r_i}. \qquad \text{Eq. S8}$$

Assuming that a steric interaction occurs between the solute and the pore walls, the solute convective $K_{c,ij}$ and diffusive $K_{d,ij}$ hindrance factors can be expressed as follows:

$$K_{c,ij} = (2-\Phi_{ij})(1+0.054\lambda_{ij} - 0.988\lambda_{ij}^2 + 0.44\lambda_{ij}^3);$$ Eq. S9

$$K_{d,ij} = 1-2.3\lambda_{ij} + 1.154\lambda_{ij}^2 + 0.224\lambda_{ij}^3.$$ Eq. S10

The Péclet number ($P_{e,ij}$) that characterizes the pore flow is defined as

$$P_{e,ij} = \frac{K_{c,ij}}{K_{d,ij} D_{s,j}} \left(\frac{r_i^2 \Delta P}{8\mu_{p,i}} \right).$$ Eq. S11

The diffusivity $D_{s,ij}$ of a solute of radius $r_{s,j}$ is calculated using the Stokes–Einstein equation:

$$D_{s,ij} = \frac{kT}{6\pi\mu_{p,i} r_{s,j}},$$ Eq. S12

where k is the Boltzmann constant and T is the temperature. The Wilke–Chang formula can be used to solve the above equation and estimate the solute's diffusivity:

$$D_{s,ij} = 7.4 \times 10^{-8} \frac{T\sqrt{\phi M_{solv}}}{\mu_{p,i} V_{m,j}^{0.6}},$$ Eq. S13

where M_{solv} is the molecular weight (M_w) of the solvent molecule, ϕ is a dimensionless solvent parameter, and $V_{m,j}$ is the solute molar volume (in cm^3 g mol^{-1}). If the rejection value $R(r)$ is a continuous function of the pore radius r, then $f_R(r)$ describes the pore-size distribution:

$$f(r) = \frac{1}{r\sqrt{2\pi b}} \exp\left[-\frac{(\log(r/r^*) + b/2)^2}{2b} \right],$$ Eq. S14

where

$$b = \log\left[1+\left(\frac{\sigma}{r^*}\right)^2\right]. \quad \text{Eq. S15}$$

To calculate the function *f(r)*, the mean pore radius (r^*) and its standard deviation (σ) must be estimated. To simplify the calculation, the distribution function was truncated to $r_{\max}$:

$$\frac{f'_R(r)}{f_R(r)} = \frac{1}{\int_0^{r_{\max}} f_R(r)dr}. \quad \text{Eq. S16}$$

The overall rejection value for pore radio size where $0 < r < r_{max}$ are then calculated as

$$R_j = \frac{\int_0^{r_{\max}} f'_R(r)r^4R(r)/\mu(r)dr}{\int_0^{r_{\max}} f'_R(r)r^4/\mu(r)dr}. \quad \text{Eq. S17}$$

By implementing the abovementioned models, the mean pore size and its standard deviation are fitted by minimizing the error.

Prior to performing the nanofiltration test, the $BioIPN^0$ was conditioned using various treatments to identify the best processing conditions. BioIPNs prepared without solvent exchange, without vacuum drying, and with neither solvent exchange nor vacuum drying were denoted as $BioIPN_{NSE}$, $BioIPN_{NVD}$, and $BioIPN_{NSEVD}$, respectively (Table S2). A linear correlation between operating pressure and acetone flux was observed (Figure S15a), indicating that $BioIPN^0$, $BioIPN_{NSE}$, and $BioIPN_{NVD}$ were neither compacted nor blocked upon the application of various levels of pressure up to 40 bar.

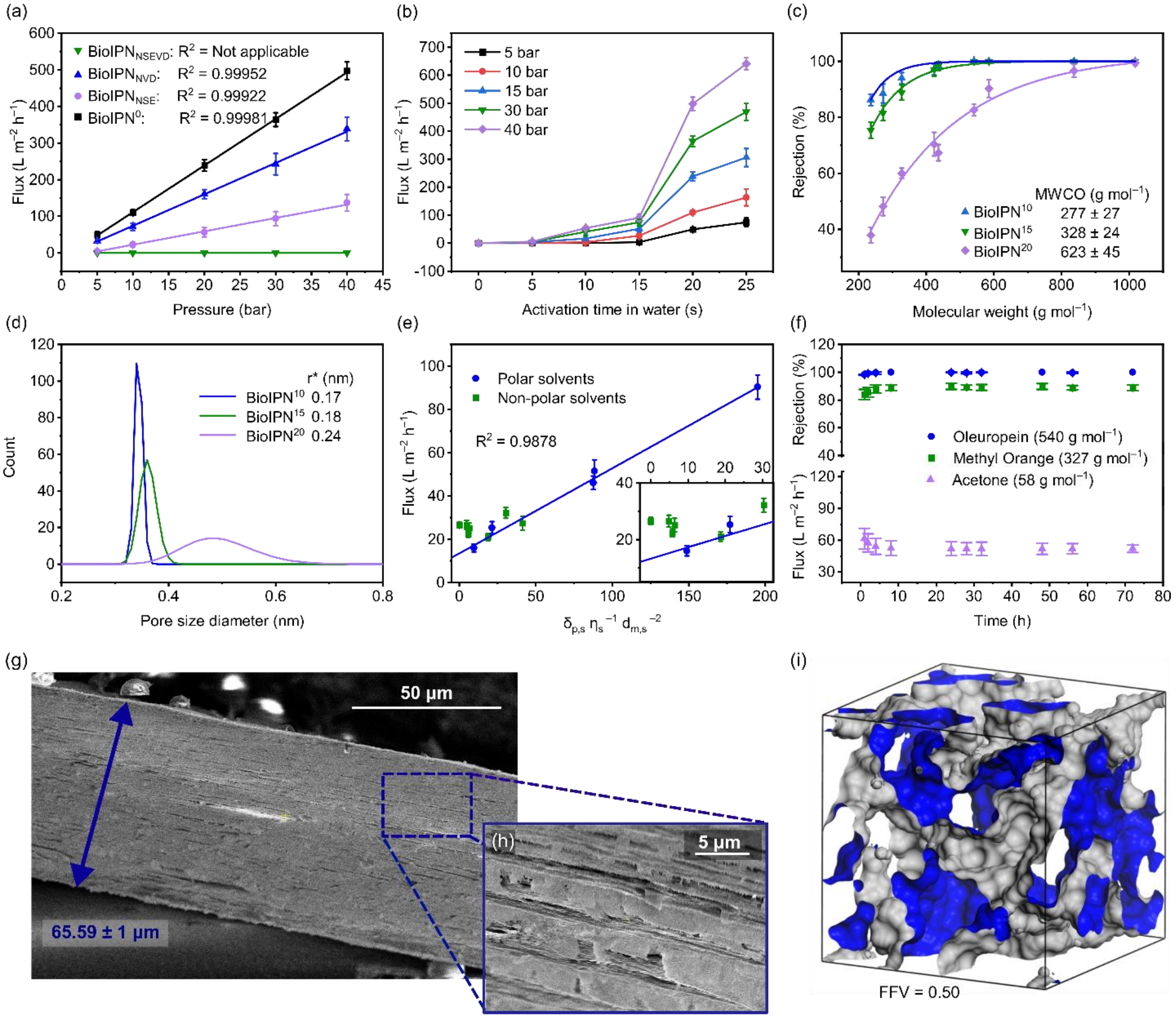

Figure S15: Nanofiltration performance of the IPN membranes BioIPN^0, BioIPN$_{NSE}$, BioIPN$_{NVD}$, and BioIPN$_{NSEVD}$. (a) Solvent flux as a function of applied pressure for membranes fabricated under different conditions. Membranes were subjected to a 15 s activation time in water. (b) Solvent flux through BioIPN^0 after being activated in water for various lengths of time, measured under multiple pressures. (c) Rejection profiles and MWCO values for BioIPN^0 after being activated in water for various lengths of time, measured at 20 bar. (d) Pore-size distribution for BioIPN after being activated in water for various lengths of time. e) Pure solvent flux profile as a function of the solubility parameter for BioIPN^{15} at 20 bar. (f) Flux and rejection profiles showing the long-term stability of BioIPN^{15}'s performance at 20 bar. Acetone was used as the solvent for nanofiltration, unless otherwise stated. (g) and (h) Cross-section cryo-SEM images and (i) simulated FFV of BioIPN^{15}.

BioIPN^0 presented superior flux under all levels of pressure investigated, followed by BioIPN$_{NVD}$ and BioIPN$_{NSE}$, whereas BioIPN$_{NSEVD}$ did not show any acetone fluxes until the pressure level reached 40 bar. These observations suggest that both processing conditions (namely

solvent exchange in ethanol and vacuum drying) played a substantial role in controlling the properties, and thus the performance of the IPN membranes. The addition of solvent exchange in ethanol to the process proved to be a more decisive factor in loosening the nanostructures than vacuum drying as $BioIPN_{NVD}$ showed higher flux than $BioIPN_{NSE}$ under all evaluated pressures.

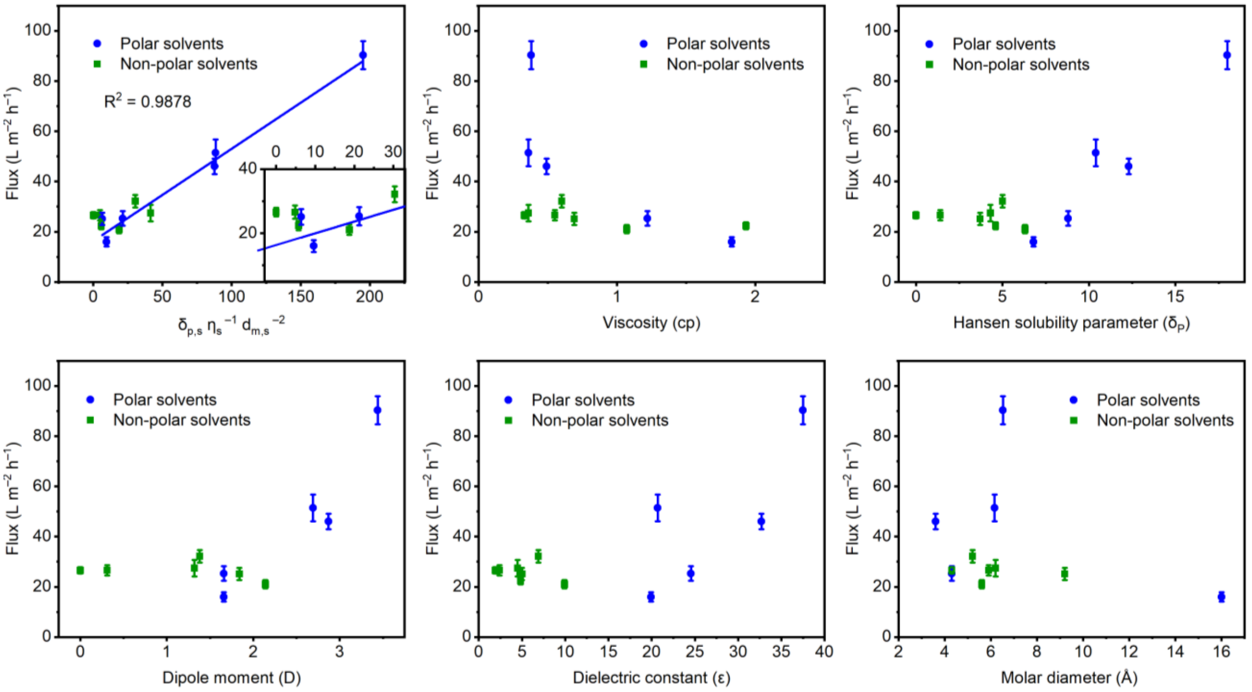

Figure S16: Pure solvent flux profiles for $BioIPN^{15}$ at 20 bar, represented as a function of (a) solubility parameter, (b) viscosity, (c) Hansen solubility parameter, (d) dipole moment, (e) dielectric constant, and (f) molar diameter. The Solvents utilized are presented in **Table S7**.

The values for the rejection of the active pharmaceutical ingredient (API) on the activated $BioIPN^{10}$, $BioIPN^{15}$, and $BioIPN^{20}$ membranes were found to be 100%, 99.89% ± 0.16%, and 98.76% ± 0.16%, respectively (Figure S17). The same values for the impurity were 60.01% ± 3.66%, 43.05% ± 3.65%, and 15.24% ± 4.36%. The uncertainty in the measured rejection and solvent flux values are reflected in the diafiltration simulation results (Figure S17h–i). Using a Monte Carlo approach, we were able to determine an uncertainty region for the descriptors of the

process performance by visualizing the 25th and 75th percentiles of the simulated concentration profiles.

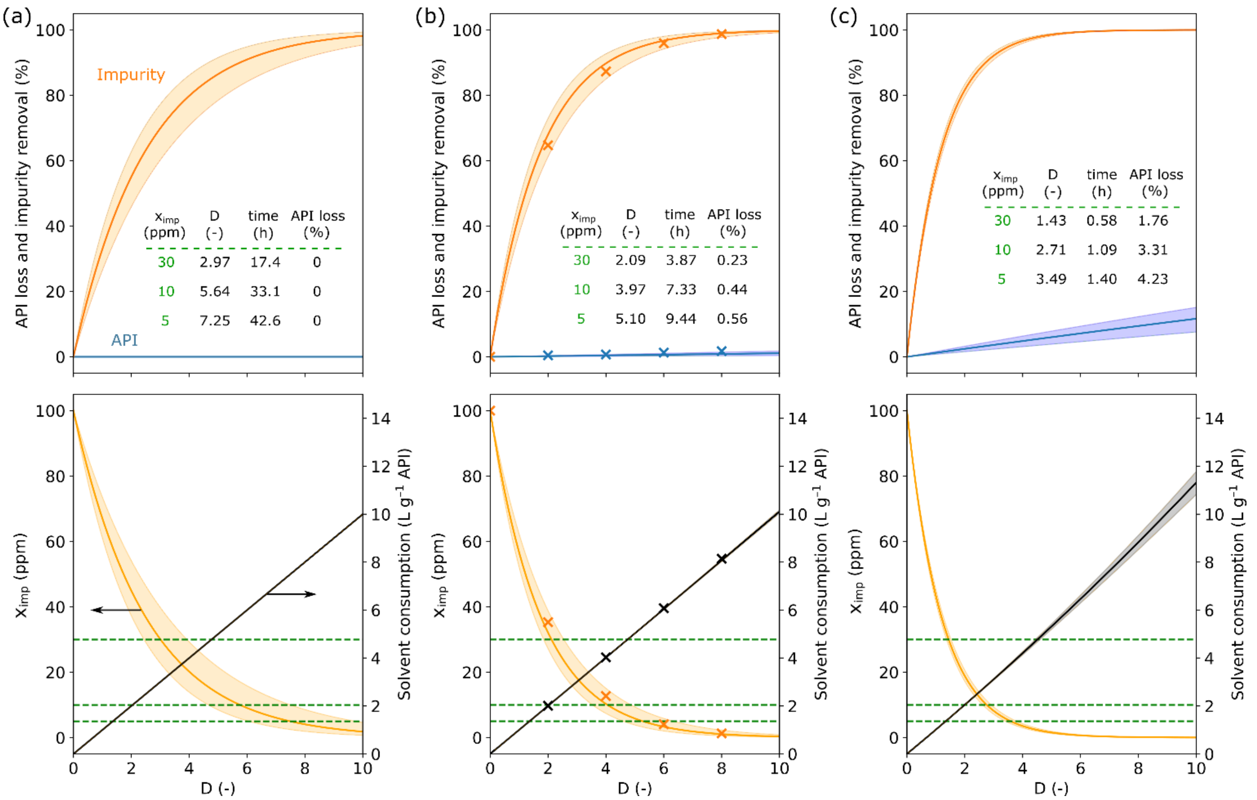

Figure S17: API loss, impurity removal, impurity ratio, and solvent consumption for a) BioIPN[10], b) BioIPN [15], and c) BioIPN [20] membranes as a function of the number of diavolumes. Measured datapoints are included for the BioIPN[15] membrane. The borders of the shaded regions correspond to the 25th and 75th percentiles of the Monte Carlo simulation results.

The impurity thresholds were calculated by dividing the threshold of toxicological concern value of 1.5 μg day^{-1} by the maximum API daily dose.[33] The high, moderate, and low daily doses for roxithromycin are 300, 150, and 50 mg day^{-1}. Diafiltration simulations demonstrated that API loss is negligible during impurity removal using the BioIPN[15] membrane. Uncertainties in the rejection and flux values can result in some deviance from the nominal levels both in API loss and solvent consumption. Nanofiltration measurements were in accordance with the simulated API and

impurity concentration trends. Nevertheless, it is important to note that the standard deviations of rejection and flux do not directly translate into the uncertainties of the performance metrics owing to the nonlinearity between membrane characteristics and process descriptors.

Table S7: Pure solvent flux determination at 20 bar for different solubility parameters.

Solvent	Solvent parameter ($\delta_{p,s}$ η_s^{-1} $d_{m,s}^{-2}$)	Viscosity (cp)	Hansen solubility parameter (δP)	Dipole moment (D)	Dielectric constant (ε)	Molar diameter (Å)	Average flux (L m^{-2} h^{-1})	Standard deviation	Polarity
Acetone	88.4	0.3	10.4	2.6	20.7	6.16	51.4	5.3	Polar
MeCN	195.0	0.4	18.0	3.4	37.5	6.52	90.2	5.6	Polar
IPA	9.6	2.4	6.8	1.6	19.92	16	15.9	1.8	Polar
Ethanol	21.3	1.1	8.8	1.6	24.55	4.3	25.3	2.8	Polar
Methanol	87.6	0.6	12.3	2.8	32.70	3.6	46.0	3.1	Polar
1,2-dichlorobenzene	18.7	1.3	6.3	2.1	9.93	5.6	21.0	1.6	Non-polar
MTBE	41.4	0.3	4.3	1.3	4.50	6.2	27.4	3.2	Non-polar
2-MeTHF	30.3	0.4	5.0	1.4	6.9	5.2	32.1	2.4	Non-polar
Heptane	0.0	0.4	0.0	0.0	1.92	4.3	26.5	1.3	Non-polar
Toluene	4.8	0.6	1.4	0.3	2.4	5.9	26.5	2.0	Non-polar
Eucalyptol	5.7	1.9	4.6	1.5	4.84	-	22.3	1.4	Non-polar
Butyl acetate	6.4	0.7	3.7	1.8	5.01	9.2	25.1	2.4	Non-polar
Variance for average flux of polar solvents:							829.7		
Variance for average flux of non-polar solvents:							13.2		

9. Molecular simulation

To further investigate the polymer packing in the membranes, density measurements were conducted. The membrane densities gradually decreased from 1.54 g cm^{-3} to 1.31 g cm^{-3} as the latex concentration in the systems increased from 0 wt% to 15 wt% (Figure S18a–d). We hypothesized that the addition of a secondary compound (latex) into the three-dimensional network of the agarose hydrogel was responsible for the introduction of structural voids, which resulted in the development of a more porous structure. The interpenetrating polymers introduced controlled defects/voids into the structure, and this promotes an increase in the FFV. As the transport phenomena through polymer membranes are governed by the free volume, manipulating the free volume within polymeric structures is essential to controlling the membrane performance. FFV simulations were performed for the models visualized in Figure S18e–h. The obtained FFV values for agarose, $BioIPN_{05}$, $BioIPN^{0}$, and $BioIPN_{15}$ (0.07, 0.12, 0.14, and 0.18, respectively) are in accordance with the density measurements of each membrane. In order to control the free volume, the formulation of IPNs was adjusted. A higher weight percentage of latex resulted in a less dense membrane.

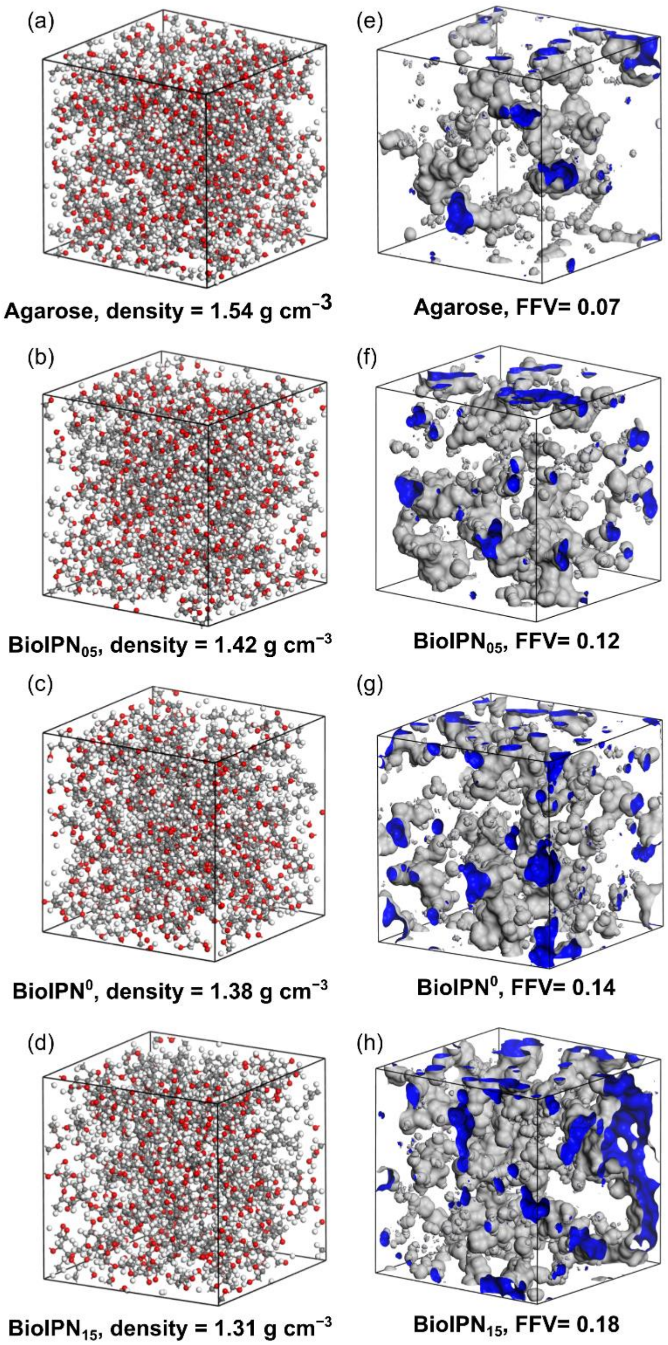

Figure S18: Molecular simulation of the polymer packing in the (a) agarose, (b) BioIPN$_{05}$, (c) BioIPN^{0}, and (d) BioIPN$_{15}$ membranes. Gray, red, and white spheres represent carbon, oxygen, and hydrogen atoms, respectively. FFV of (e) agarose, (f) BioIPN$_{05}$, (g) BioIPN^{0}, and (h) BioIPN$_{15}$.

To accurately predict the new density of the swollen $BioIPN^0$ after immersion in water, a simulation in virtual space was carried out. To simplify our calculations, we assumed an isotropic swelling[8,9] of $BioIPN^0$ upon contact with water occurred during the activation time of 15 s.

For a fixed lattice parameter of a_i = 30 Å^3 in Materials Studio software, the mass W_i initially placed in the cubic cell was calculated using the Archimedes density ρ_i = 1.38 g cm^{-3} determined for the dry $BioIPN^0$.

$$W_i\ (g) = \rho_i(\mathrm{g\ Å^{-3}}) * a_i(\mathrm{Å^3}) \qquad \text{Eq. S18}$$

yielding W_i = 4.14 × 10^{-35} g. The lattice parameter a_f of the swollen system was determined based on the thicknesses T_i = 34.71 μm of the dry $BioIPN^0$ and T_f = 65.59 μm of the swollen $BioIPN^{15}$, both dry and swollen membranes can be observed in Figure S19b–c.

$$a_f(\mathrm{Å^3}) = \frac{a_i\ (\mathrm{Å^3}) * T_f\ (\mathrm{\mu m})}{T_i\ (\mathrm{\mu m})} \qquad \text{Eq. S19}$$

where a_f = 56.69 Å^3. As the volume of expansion would be occupied by voids in this case, the density (ρ_f) of the activated $BioIPN^{15}$ was then derived for a new predicted cell parameter (a_f, in Figure S19a) as follows:

$$\rho_f(g\ cm^{-3}) = \frac{W_i\ (\mathrm{g}) * 10^{36}\ (\mathrm{Å^3})}{a_f\ (\mathrm{Å^3}) * \mathrm{cm^3}} \qquad \text{Eq. S20}$$

Finally, the density (ρ_f) of the swollen IPN was determined to be 0.73 g cm^{-3} and a new simulation was performed to identify the FFV (0.50) of BioIPN[15] at the moment of nanofiltration in the OSN cross-flow filtration system (Figure S20).

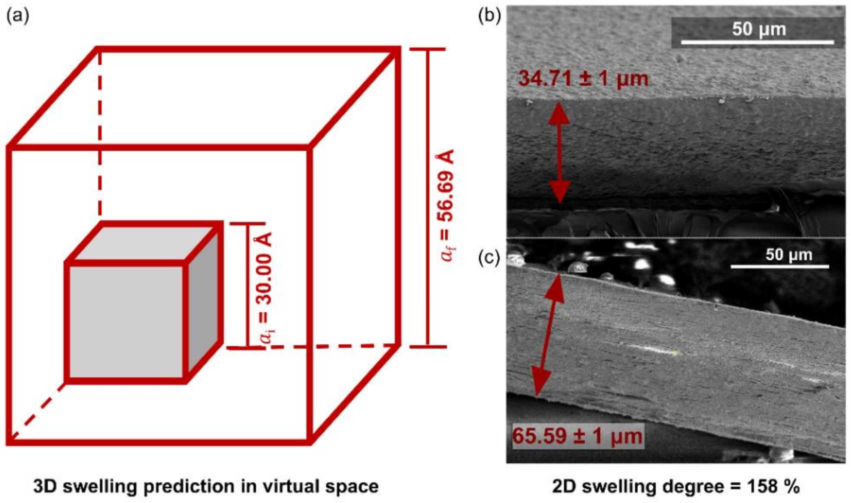

Figure S19: Schematic drawing of BioIPN^0's: (a) predicted 3D swelling in virtual space according to the 2D swelling degree found via (b) SEM and (c) cryo-SEM.

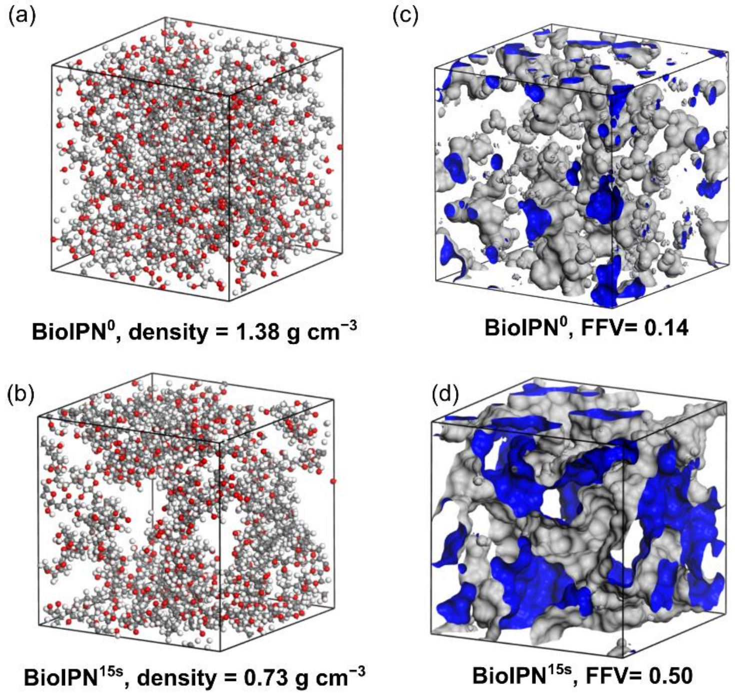

Figure S20: Molecular simulation of the (a, b) polymer packing and (c, d) FFV in $BioIPN^0$ before and after 15 s activation in water.

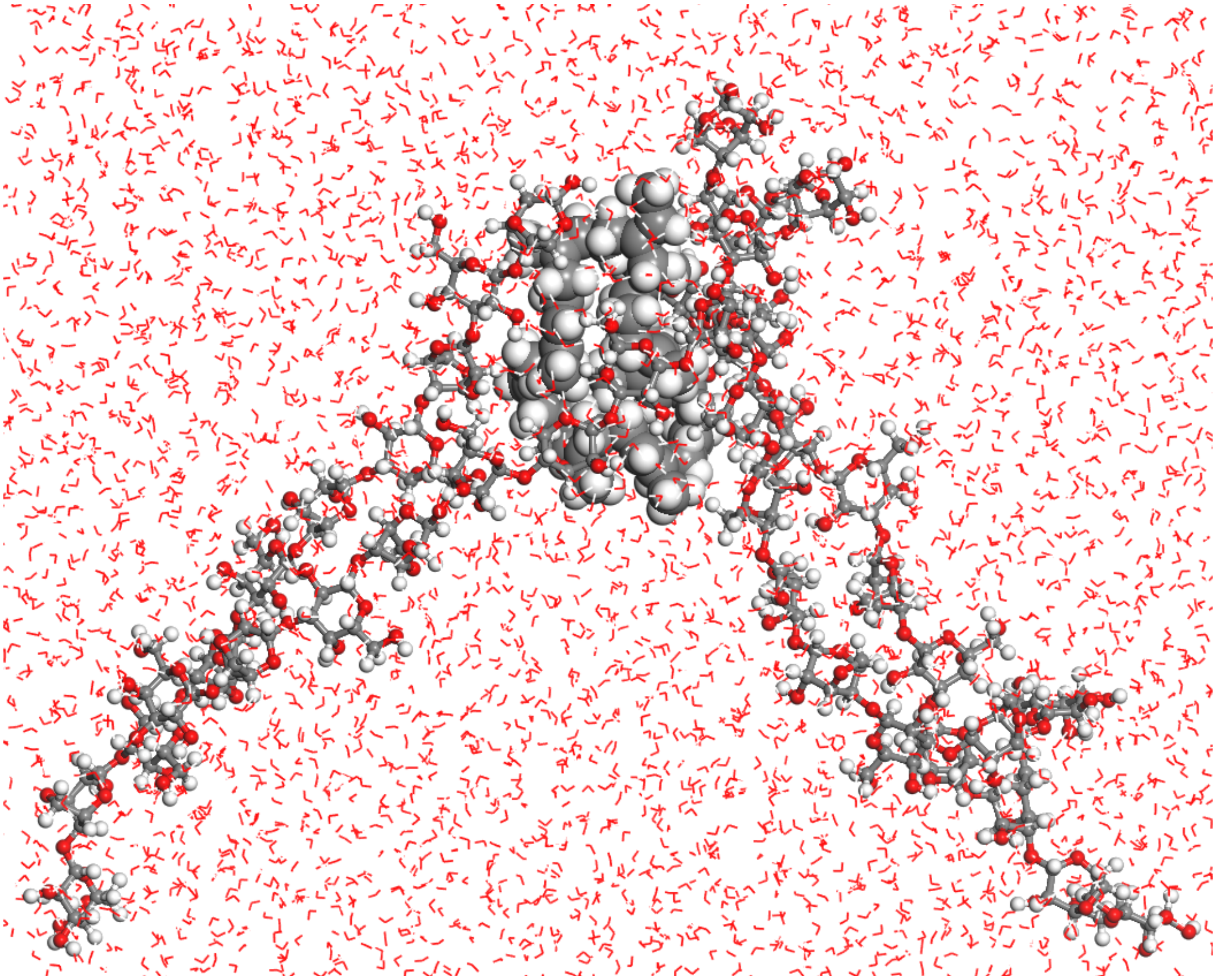

Figure S21: Four chains of agarose and one chain of latex in the presence of water. Water was chosen as solvent because it was used as the medium for membrane preparation.

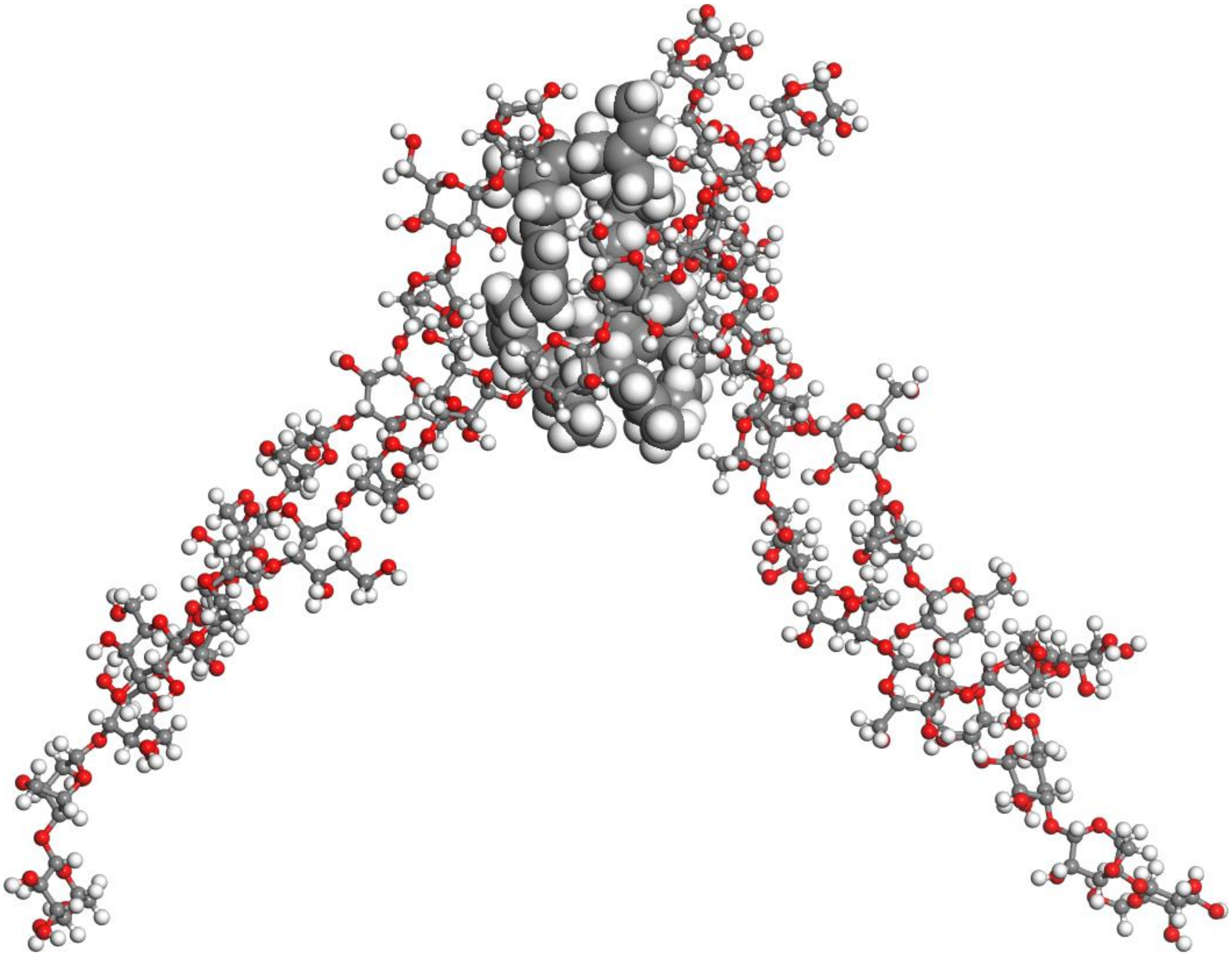

Figure S22: Same as Fig. S21. The water molecules are not shown for clarity.

Table S8: Permeance and MWCO of the BioIPN membrane compared to other IPNs and tightest membranes.

Type	Solvent	Permeance ($L\ m^{-2}\ h^{-1}\ bar^{-1}$)	MWCO ($g\ mol^{-1}$)	References
BioIPN	Acetone	1.7	227 – 623	this study
IPN	DMSO	0.2	190 – 850	[10]
Semi-IPN	Water	3	3000	[11]
CMS-fullerenol	Acetone	1.85 – 2.26	230 – 356	[12]
CMS	Acetone	0.1 – 1.8	122 – 291	[13]
M3: 24 wt% PI	Toulene	1.66	250	[14]
M4: 26 wt% PI	Toulene	1.0	250	[14]
M1 (DMF-dioxane-1:3)	Toulene	3.6	<200	[15]
M2 (DMF-dioxane-1:1)	Toulene	-	420	[15]
StarMem 122	Toluene	1.16	220	[16]
StarMem240	Toulene	2.05	400	[16]
PBI-H2SO4	Methanol	3.5	500	[17]
X-PBI	IPA	5.8	440	[18]
SPEEK (10 wt%)	THF	0.42–0.52	395-495	[19]
SPEEK (12 wt%)	THF	0.18–0.40	295-395	[19]
Teflon AF2400/PE	Acetone	1.15	150	[20]

References

(1) Canevarolo, S. V. Polymer Molecular Structure. In *Polymer Science*; Carl Hanser Verlag GmbH & Co. KG, 2019; pp 13–53. https://doi.org/10.3139/9781569907269.002.

(2) Altman, R. F. A. Natural Coagulation of Hevea Latex. *Rubber Chem. Technol.* **1947**, *20* (4), 1124–1132. https://doi.org/10.5254/1.3543323.

(3) Ng, J. W.; Othman, N.; Yusof, N. H. Various Coagulation Techniques and Their Impacts towards the Properties of Natural Rubber Latex from Hevea Brasiliensis — a Comprehensive Review Related to Tyre Application. *Ind. Crops Prod.* **2022**, *181*, 114835. https://doi.org/10.1016/j.indcrop.2022.114835.

(4) Kim, S.-Y.; Seong, H. Modulation of Physicochemical Properties of Magnetic Agarose Microspheres by Hydrolysis-Suppressive Sequential Crosslinking. *Colloids Surf. Physicochem. Eng. Asp.* **2021**, *630*, 127607. https://doi.org/10.1016/j.colsurfa.2021.127607.

(5) Liu, J.; Xu, Q.; Jiang, J. A Molecular Simulation Protocol for Swelling and Organic Solvent Nanofiltration of Polymer Membranes. *J. Membr. Sci.* **2019**, *573*, 639–646. https://doi.org/10.1016/j.memsci.2018.12.035.

(6) Balaji, K. R.; Hardian, R.; Kumar, V. G. D.; Viswanatha, R.; Kumar, S.; Kumar, S.; Singh, A.; Santosh, M. S.; Szekely, G. Composite Nanofiltration Membrane Comprising One-Dimensional Erdite, Two-Dimensional Reduced Graphene Oxide, and Silkworm Pupae Binder. *Mater. Today Chem.* **2021**, *22*, 100602. https://doi.org/10.1016/j.mtchem.2021.100602.

(7) Karan, S.; Jiang, Z.; Livingston, A. G. Sub–10 Nm Polyamide Nanofilms with Ultrafast Solvent Transport for Molecular Separation. *Science* **2015**, *348* (6241), 1347–1351. https://doi.org/10.1126/science.aaa5058.

(8) Duque, C. M.; Chen, B. G.; Santangelo, C. D. Distortion-Controlled Isotropic Swelling: Numerical Study of Free Boundary Swelling Patterns. *Soft Matter* **2019**, *15* (24), 4890–4897. https://doi.org/10.1039/C9SM00392D.

(9) Krauklis, A. E.; Gagani, A. I.; Echtermeyer, A. T. Prediction of Orthotropic Hygroscopic Swelling of Fiber-Reinforced Composites from Isotropic Swelling of Matrix Polymer. *J. Compos. Sci.* **2019**, *3* (1), 10. https://doi.org/10.3390/jcs3010010.

(10) Zhao, D.; Kim, J. F.; Ignacz, G.; Pogany, P.; Lee, Y. M.; Szekely, G. Bio-Inspired Robust Membranes Nanoengineered from Interpenetrating Polymer Networks of Polybenzimidazole/Polydopamine. *ACS Nano* **2019**, *13* (1), 125–133. https://doi.org/10.1021/acsnano.8b04123.

(11) Nozad, E.; Poursattar Marjani, A.; Mahmoudian, M. A Novel and Facile Semi-IPN System in Fabrication of Solvent Resistant Nano-Filtration Membranes for Effective Separation of Dye Contamination in Water and Organic Solvents. *Sep. Purif. Technol.* **2022**, *282*, 120121. https://doi.org/10.1016/j.seppur.2021.120121.

(12) Hardian, R.; Abdulhamid, Mahmoud. A.; Szekely, G. Nanodomain Control in Carbon Molecular Sieve Membranes via Nanomaterial Footprinting. *Small Sci. n/a* (n/a), 2300162. https://doi.org/10.1002/smsc.202300162.

(13) Abdulhamid, M. A.; Hardian, R.; Szekely, G. Carbon Molecular Sieve Membranes with Integrally Skinned Asymmetric Structure for Organic Solvent Nanofiltration (OSN) and Organic Solvent Reverse Osmosis (OSRO). *Appl. Mater. Today* **2022**, *28*, 101541. https://doi.org/10.1016/j.apmt.2022.101541.

(14) See-Toh, Y. H.; Ferreira, F. C.; Livingston, A. G. The Influence of Membrane Formation Parameters on the Functional Performance of Organic Solvent Nanofiltration Membranes. *J. Membr. Sci.* **2007**, *299* (1), 236–250. https://doi.org/10.1016/j.memsci.2007.04.047.

(15) See-Toh, Y. H.; Silva, M.; Livingston, A. Controlling Molecular Weight Cut-off Curves for Highly Solvent Stable Organic Solvent Nanofiltration (OSN) Membranes. *J. Membr. Sci.* **2008**, *324* (1), 220–232. https://doi.org/10.1016/j.memsci.2008.07.023.

(16) Darvishmanesh, S.; Degrève, J.; Van der Bruggen, B. Physicochemical Characterization of Transport in Nanosized Membrane Structures. *ChemPhysChem* **2010**, *11* (2), 404–411. https://doi.org/10.1002/cphc.200900641.

(17) Asadi Tashvigh, A.; Chung, T.-S. Robust Polybenzimidazole (PBI) Hollow Fiber Membranes for Organic Solvent Nanofiltration. *J. Membr. Sci.* **2019**, *572*, 580–587. https://doi.org/10.1016/j.memsci.2018.11.048.

(18) Davood Abadi Farahani, M. H.; Chung, T.-S. A Novel Crosslinking Technique towards the Fabrication of High-Flux Polybenzimidazole (PBI) Membranes for Organic Solvent Nanofiltration (OSN). *Sep. Purif. Technol.* **2019**, *209*, 182–192. https://doi.org/10.1016/j.seppur.2018.07.026.

(19) da Silva Burgal, J.; Peeva, L.; Marchetti, P.; Livingston, A. Controlling Molecular Weight Cut-off of PEEK Nanofiltration Membranes Using a Drying Method. *J. Membr. Sci.* **2015**, *493*, 524–538. https://doi.org/10.1016/j.memsci.2015.07.012.

(20) Shi, G. M.; Chung, T.-S. Teflon AF2400/Polyethylene Membranes for Organic Solvent Nanofiltration (OSN). *J. Membr. Sci.* **2020**, *602*, 117972. https://doi.org/10.1016/j.memsci.2020.117972.
